# Serum Proteomic Signatures of Rheumatoid Arthritis Risk and Response: Analysis of a Rheumatoid Arthritis Interception Trial

**DOI:** 10.1002/art.70082

**Published:** 2026-02-17

**Authors:** Marianna Jasenecova, Carl Coyle, Alina Mihalovits, Sarah Ryan, Elizabeth Pook, Esperanza Perucha, Gabrielle Harker, Melody Chin, Sam Norton, Andrew P. Cope

**Affiliations:** ^1^ Centre for Rheumatic Diseases King's College London London United Kingdom

## Abstract

**Objective:**

Our study objective was to identify serum protein signatures associated with progression to rheumatoid arthritis (RA) and response to abatacept in at‐risk individuals.

**Methods:**

A total of 440 serum samples from 118 APIPPRA (Arthritis Prevention In the Preclinical Phase of RA with Abatacept) study participants were selected from baseline to RA onset for 46 progressors of RA or to study end for 72 participants who did not develop RA. Samples were analyzed using the SomaScan 7k assay platform. Differential expression analysis was assessed by progression to RA (three pre‐RA time intervals to RA, progressors of RA vs nonprogressors, baseline to RA), and by treatment allocation (abatacept vs placebo). Risk and response signatures were identified in the full 7k panel and two prespecified subpanels defined as Inflammatory Mediators and Adaptive Immune Cell panel.

**Results:**

We observed significant changes in 80 proteins (68 down‐regulated and 12 up‐regulated) occurring between RA onset and 6 to 24 months before developing disease. Progression to RA was associated with increased levels of acute‐phase reactants SAA1 and SAA2 and reductions in CTLA4, when compared to nonprogressors at the end of treatment. Two up‐regulated proteins (CTLA4 and CD86) and seven down‐regulated proteins (CXCL13, FCRL4, FCER2, CCL21, LTA|LTB, FDCSP, and IL22RA2) were observed in participants receiving abatacept compared to placebo regardless of RA outcome.

**Conclusion:**

Protein signatures dominated by acute‐phase proteins define progression to RA, whereas changes associated with abatacept therapy highlight potential mechanisms of treatment response. Such signatures provide a better understanding of the immune landscape of the at‐risk phase, opening up the possibility of new treatment modalities for RA prevention.

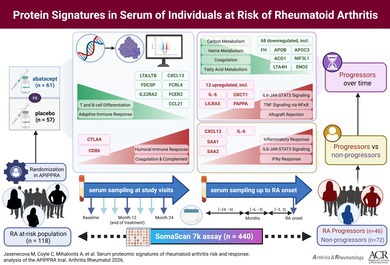

## Graphical abstract

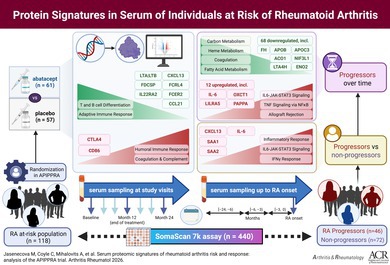

## INTRODUCTION

There has been significant progress in understanding at‐risk states in immune‐mediated inflammatory disease. In rheumatoid arthritis (RA), these at‐risk states have prompted large prospective cohort studies and analysis of trajectories that have helped define more consistent rates of progression over time.^1^, ^2^, ^3^, ^4^, ^5^, ^6^ These in turn have supported efforts to refine risk stratification where risk phenotypes can be applied to the clinical setting.^7^ The recent development of RA risk scores based upon clinical symptoms such as clinically suspect arthralgia and the presence of serum autoantibodies, with or without imaging, have provided an experimental framework for the design of randomized clinical trials aimed at preventing or at least delaying the onset of clinically apparent arthritis and classifiable disease. The results of several interception trials have been published, demonstrating the value of such an approach, as well as providing evidence for treatment of symptoms on drug, and both prevention of progression and delay depending on the therapeutic intervention.^4^, ^8^, ^9^, ^10^, ^11^, ^12^, ^13^, ^14^, ^15^

These trials highlight many of the challenges that need to be addressed before interception can be delivered in the routine clinical setting. First, many individuals deemed to be at high risk do not progress to RA, despite years of follow‐up.^2^, ^5^, ^16^, ^17^, ^18^ Second, besides symptoms and autoantibodies, we lack knowledge of specific immune inflammatory mechanisms associated with the at‐risk phenotype.^19^ Third, we have yet to identify the signatures associated with drug response and favorable clinical outcomes. Applying a SomaScan 7k aptamer‐based proteomics platform,^20^ we set out to examine serum samples from at‐risk individuals enrolled in the APIPPRA randomized placebo‐controlled clinical trial and define signatures associated with progression to disease and response to the study drug abatacept.

## METHODS

### Trial design and study participants

**Figure 1:**
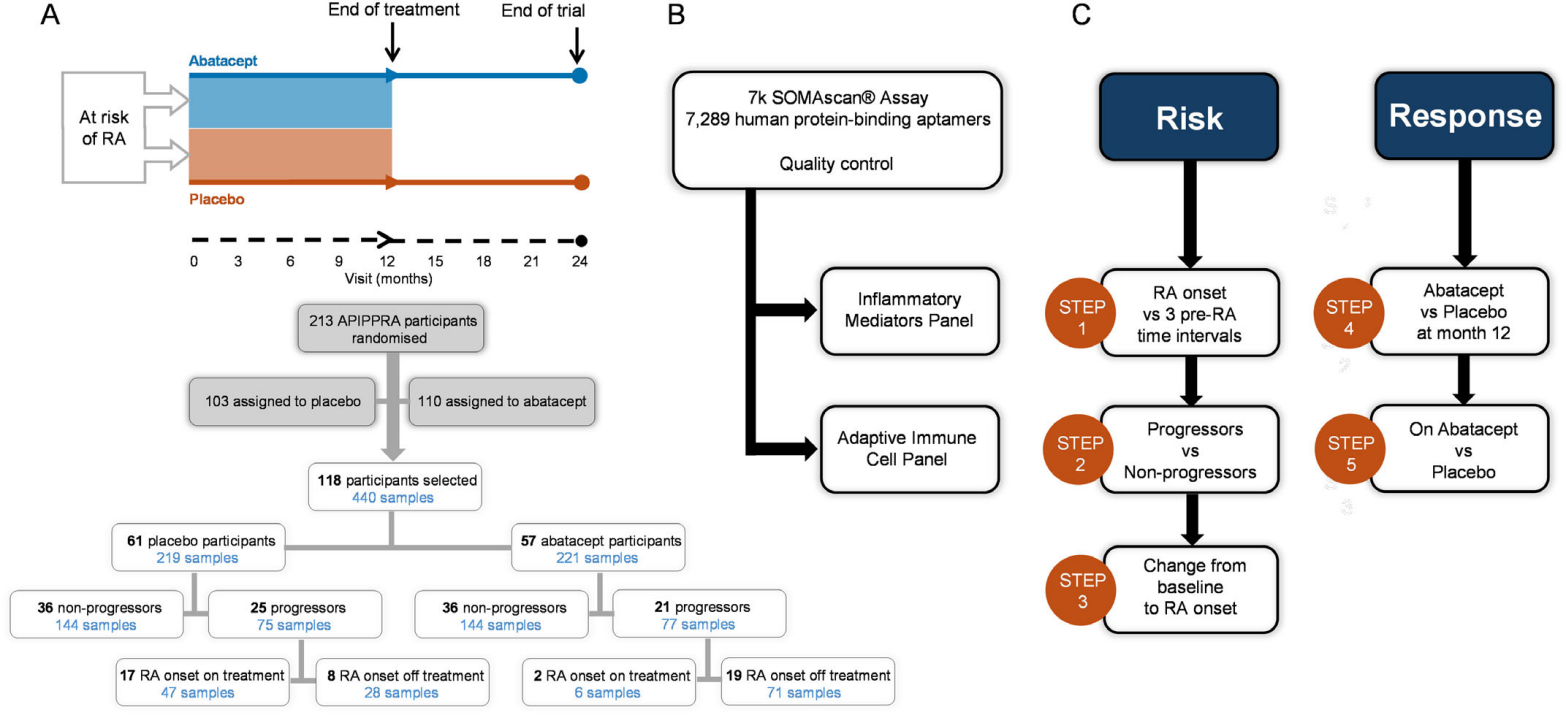
Study workflow and Sample acquisition stratified by trial arm and outcome. (A) APIPPRA study participants received 12 months of treatment (abatacept or placebo) before additional 12 months of follow‐up. Serum samples were collected every three months from baseline to 24 months, processed, and stored at −80°C before analysis. (B) Samples were analyzed on the 7k SomaScan aptamer‐based platform. (C) Five steps to investigate risk and response biomarkers. “On abatacept” refers to samples collected from participants receiving abatacept treatment during the first year, excluding baseline. APIPPRA, Arthritis Prevention In the Preclinical Phase of RA with Abatacept; RA, rheumatoid arthritis.

The APIPPRA study, registered with EudraCT 2013‐003413‐18, was a phase 2b multicenter placebo‐controlled clinical trial conducted in the United Kingdom and the Netherlands.^9^, ^21^ Participants at risk of RA were defined by inflammatory joint pain and either being positive for antibodies to citrullinated protein antigens (ACPA) and rheumatoid factor or having ACPA more than three times upper limit of normal. Key exclusion criteria included previous episodes of clinical synovitis and use of glucocorticoids or disease‐modifying antirheumatic drugs (DMARDs). Participants were randomly assigned to weekly subcutaneous injections of 125 mg abatacept or placebo for 12 months, after which treatment was stopped, with 12 months of further follow‐up (Figure 1A). The primary endpoint was time to RA development according to American College of Rheumatology and EULAR 2010 criteria^22^ or clinical synovitis in three or more joints, whichever occurred first.

### Sample selection

A total of 440 samples were selected from 118 APIPPRA study participants, of whom 61 were randomized to the placebo arm and 57 to the abatacept group (Figure 1A). With exception of the allocated treatment, selected participants were not prescribed any DMARDs or glucocorticoids before meeting classification criteria for RA and were sustained fully compliant with the study medication.

Participants were categorized into three groups based on RA progression status and treatment allocation. The nonprogressors group (n = 72, including 36 each in abatacept and placebo arms) had samples acquired at four time points: baseline, end of treatment (month 12), month 15, and end of study (month 24). Progressors were subdivided by treatment group: placebo progressors (n = 25) and abatacept progressors (n = 21). For progressors, samples were acquired at baseline, pre‐endpoint visit (before RA onset), and endpoint visit (RA onset), with additional time points collected for those progressing after the 12‐month dosing period. Overall, 152 samples from progressors were analyzed in four time intervals: 47 samples in 6 to 24 months before RA onset, 19 samples in 3 to 6 months, 40 samples in up to 3 months to RA onset, and 46 samples at RA onset. These compared with 288 samples from nonprogressors, yielding a total of 440 samples for analysis.

### 7k SomaScan assay and bespoke panels

Participants’ serum was analyzed using the SomaScan assay v4.1^23^ comprising 7,289 SOMAmer (Slow Off‐Rate Modified Aptamer) human protein‐binding reagents (hereafter, “7k panel”). To understand how protein relevant to specific pathways changed, we defined two prespecified bespoke subpanels, comprising 479 inflammatory mediators and 373 associated with adaptive immune responses (hereafter, “Inflammatory Mediators” and “Adaptive Immune Cell” panels) (Figure 1B). Protein levels were expressed as arbitrary relative fluorescence units (RFU), considered directly proportional to the amount of the target epitope present in the sample. See Supplementary Materials for protein mapping and quality control assessment.

### Statistical analysis

Statistical analyses were performed using R version 4.3.1 (2023‐06‐16 ucrt),^24^ with SOMA adat file processing conducted using the SomaDataIO R package. Proteomics data were merged with clinical trial databases to incorporate relevant additional clinical variables. All SOMAmer reagents were expressed in arbitrary RFU units and log2‐transformed for analysis. Unscheduled visits were assigned to the monthly time point within a four‐week window, and four samples, acquired 29 to 30 months postrandomization due to the COVID‐19 pandemic, were assigned to the 24‐month time point. Further details are provided in the statistical analysis plan (Supplementary Data).

#### Differential expression analysis

Differential expression analysis employed multiple statistical approaches depending on the specific comparison (Figure 1C). Temporal changes preceding RA onset were examined using a linear mixed‐effects models with fixed effects for pre‐RA time intervals (RA onset, 0–3 months, 3–6 months, 6–24 months pre‐RA) and treatment (on abatacept, off abatacept, placebo) and random per‐subject intercepts (Figure 1C, step 1). Linear mixed‐effects models with random per‐subject levels and fixed effects for treatment (on abatacept, off abatacept, and placebo) and categorical time point, accounting for baseline values, were used to test differences between participants receiving abatacept and placebo (Figure 1C, step 5). “On abatacept” refers to samples collected from participants receiving abatacept treatment during the first year, excluding baseline. For independent group comparisons, Welch's *t*‐test with unequal variances was used (Figure 1C, step 2 and 4), or paired *t* tests for within‐subject comparisons (Figure 1C, step 3).

#### False discovery rate and fold change

*P* values were adjusted by Benjamini‐Hochberg false discovery rate (FDR) method^25^ to control for Type I error across multiple hypothesis tests, applied independently within each subpanel. An adjusted *P* value cutoff of 0.05 was applied to identify significant differential expressed proteins. Log2 fold change (log2FC) was computed as the difference in means of log2‐transformed protein expression values between comparison groups. For independent comparisons, log2FC represented mean differences between groups, whereas for paired analyses, log2FC reflected within‐subject changes. Contrasts in estimated means from mixed‐effects models were calculated using the R package emmeans. Because FC in SomaScan signal may not represent a FC in protein concentration when comparing different reagents, FC thresholds were not applied.

#### Sensitivity and correlation analyses

Strong monotonic associations between differentially expressed proteins were assessed using Spearman's rank correlation coefficients, with absolute correlation coefficients >0.7 visually assessed using correlation matrices and chord diagrams generated with the circlize R package. Sensitivity analyses were conducted to investigate differentially expressed proteins with unadjusted *P* value < 0.05, with potential outliers examined and excluded where applicable.

#### Pathway analysis and Gene Ontology

Kyoto Encyclopedia of Genes and Genomes (KEGG) pathway analysis and Gene Ontology (GO) enrichment analysis were performed separately for up‐regulated and down‐regulated protein sets defined as having adjusted *P* value of < 0.05 for more than 10 proteins, or unadjusted *P* value of < 0.01. For detailed information, see the supplementary documents. Gene Set Enrichment Analysis (GSEA) was conducted on the combined set of up‐regulated and down‐regulated proteins, using an unadjusted *P* < 0.05 to define inclusion. Proteomic data will be shared upon reasonable request.

## RESULTS

Baseline demographic characteristics of the 118 APIPPRA participants included in the analysis are shown in Table 1. Mean ± SD age was 48.1 ± 11.1 years, 79% of participants (93 of 118) were female, 79% (93 of 118) were of White ethnicity, and 59% (70 of 118) were current or previous smokers. Baseline features were balanced between arms.

**Table 1:** Baseline characteristics of APIPPRA trial participants included in the study*

	Placebo (n = 61)	Abatacept (n = 57)	Total (N = 118)
Age, mean ± SD, y	48.1 (10.7)	48.1 (11.7)	48.1 (11.1)
Sex			
Female	50 (82)	43 (75)	93 (79)
Male	11 (18)	14 (25)	25 (21)
Ethnicity			
White	48 (79)	45 (79)	93 (79)
Asian	6 (10)	6 (11)	12 (10)
Black	4 (7)	5 (9)	9 (8)
Mixed	2 (3)	1 (2)	3 (3)
Other	1 (2)	0 (0)	1 (1)
Smoking status			
Current	12 (20)	11 (19)	23 (19)
Previous	26 (43)	21 (37)	47 (40)
Never	23 (38)	25 (44)	48 (41)
RA outcome status			
Nonprogressors	36 (59)	36 (63)	72 (61)
Progressors	25 (41)	21 (37)	46 (39)

* Data are in n (%) unless otherwise indicated. APIPPRA, Arthritis Prevention In the Preclinical Phase of RA with Abatacept; RA, rheumatoid arthritis.

### Longitudinal changes in risk signatures among progressors of RA

**Figure 2:**
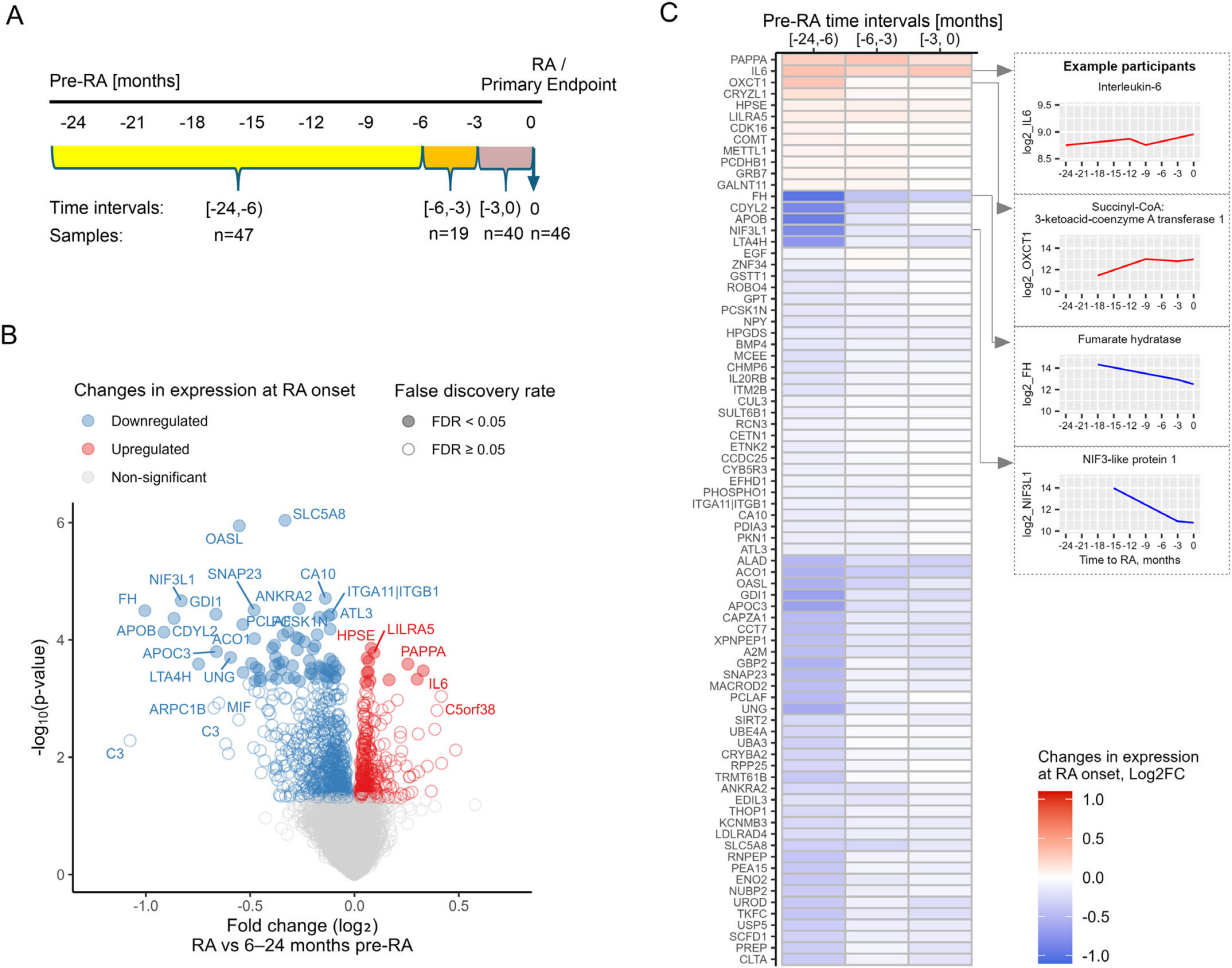
Changes in protein expression from the at‐risk phase to RA onset. (A) The at‐risk phase was divided into three time intervals: 6 to 24 months, 3 to 6 months, and up to 3 months before RA onset. (B) Differentially expressed proteins in serum at RA onset compared to 6 to 24 months before onset are presented as a volcano plot. (C) The heatmap illustrates the log_2_ fold changes across the three pre‐RA intervals for 80 differentially expressed proteins at 6 to 24 months. Longitudinal distributions of IL‐6, OXCT1, FH, and NIF3L1 show protein trajectories for individual participants, each line connecting three to four observations. FC, fold change; FDR, false discovery rate; RA, rheumatoid arthritis.

We first set out to examine changes in expression of serum proteins during the period from the at‐risk state to RA onset to understand disease development over time (Figure 1C, steps 1–3). For the 46 participants who progressed to RA, we compared samples from onset with three time intervals (≤3 months, 3–6 months and 6–24 months) before disease onset (Figure 2A), adjusting for treatment arm. Significant changes from 6 to 24 months pre‐RA to RA onset were defined for 12 up‐regulated proteins at RA onset, including interleukin 6 (IL‐6), OXCT1, PAPPA, and LILRA5, and 68 down‐regulated proteins, including FH, APOB, CDYL2, and NIF3L1, in the 7k panel (Figure 2B). A complete list of 80 identified proteins is provided in Supplementary Table S1. FC for expressed proteins at RA onset showed 40% to 50% reductions in FH, APOB, CDYL2, NIF3L1, and LTA4H and 19% to 26% increases in IL‐6, OXCT1, and PAPPA compared to 6 to 24 months pre‐RA (Figure 2C). The most significant pathway associated with down‐regulated proteins was carbon metabolism (KEGG, identifier hsa01200). There were no pathways associated with up‐regulated proteins. Down‐regulated fatty acid metabolism, heme metabolism, and coagulation GSEA pathways were identified for expressed proteins at RA onset compared to 6 to 24 months pre‐RA (Supplementary Figure S1). Out of the 80 differentially expressed proteins, strong (correlation coefficient > 0.7) monotonic relationships were observed among 47 down‐regulated and 4 up‐regulated proteins (Supplementary Figure S2). Strong negative associations were seen between down‐regulated OASL, GDI1, PKN1, and OXCT1 (coefficient −0.75). The strongest positive associations were observed between down‐regulated proteins: OASL and SLC5A8 (coefficient 0.94), OASL and ANKRA2 (coefficient 0.91), or APOB and SNAP23 (coefficient 0.91) across all study visits.

Next, we ran the same analysis in subpanels of 479 inflammatory mediators and 373 adaptive immune cells to reduce multiple testing burden and increase statistical power. FDR was controlled separately within each panel using the Benjamini‐Hochberg method. In the Inflammatory Mediators panel there were three down‐regulated proteins—IL20RB, BMP4, and EGF—and one up‐regulated protein—IL‐6 (Supplementary Figure S3A). No differentially expressed proteins were observed in the Adaptive Immune Cell panel (Supplementary Figure S3B).

Comparing proteomic changes at RA onset with three to six months pre‐RA, we found no differentially expressed proteins in the 7k panel (Supplementary Figure S4A). In the Inflammatory Mediators panel, we observed up‐regulated SAA2 and C1QTNF1 at RA onset (Supplementary Figure S4B) but no down‐regulated differentially expressed proteins. In the Adaptive Immune Cell panel, we identified a single down‐regulated protein, GPNMB (Supplementary Figure S4C). SAA2, C1QTNF1, and GPNMB were not statistically significant in the 6 to 24 months pre‐RA. In addition to down‐regulated fatty acid metabolism pathway, we observed up‐regulated inflammatory response and allograft rejection GSEA pathways associated with expressed proteins at RA onset compared to three to six months pre‐RA (Supplementary Figure S5).

Next, we studied the three‐month period before RA onset and did not observe any differentially expressed proteins in the 7k or bespoke panels (Supplementary Figure S6A–C). In the sensitivity analysis, we observed 234 down‐regulated and 83 up‐regulated proteins from the 7k panel (Supplementary Figure S6A), with unadjusted *P* value < 0.05, but none passed the FDR correction. Notable among these were acute‐phase proteins IL‐6, SAA1, and SAA2, as well as CXCL13. Down‐regulated heme metabolism, up‐regulated IL6 JAK STAT3 signaling, and tumor necrosis factor‐α (TNF‐α) signaling via NF‐κB GSEA pathways were associated with expressed proteins at RA onset compared to zero to three months pre‐RA (Supplementary Figure S7).

### Risk signatures distinguishing at‐risk individuals and progressors of RA

**Figure 3:**
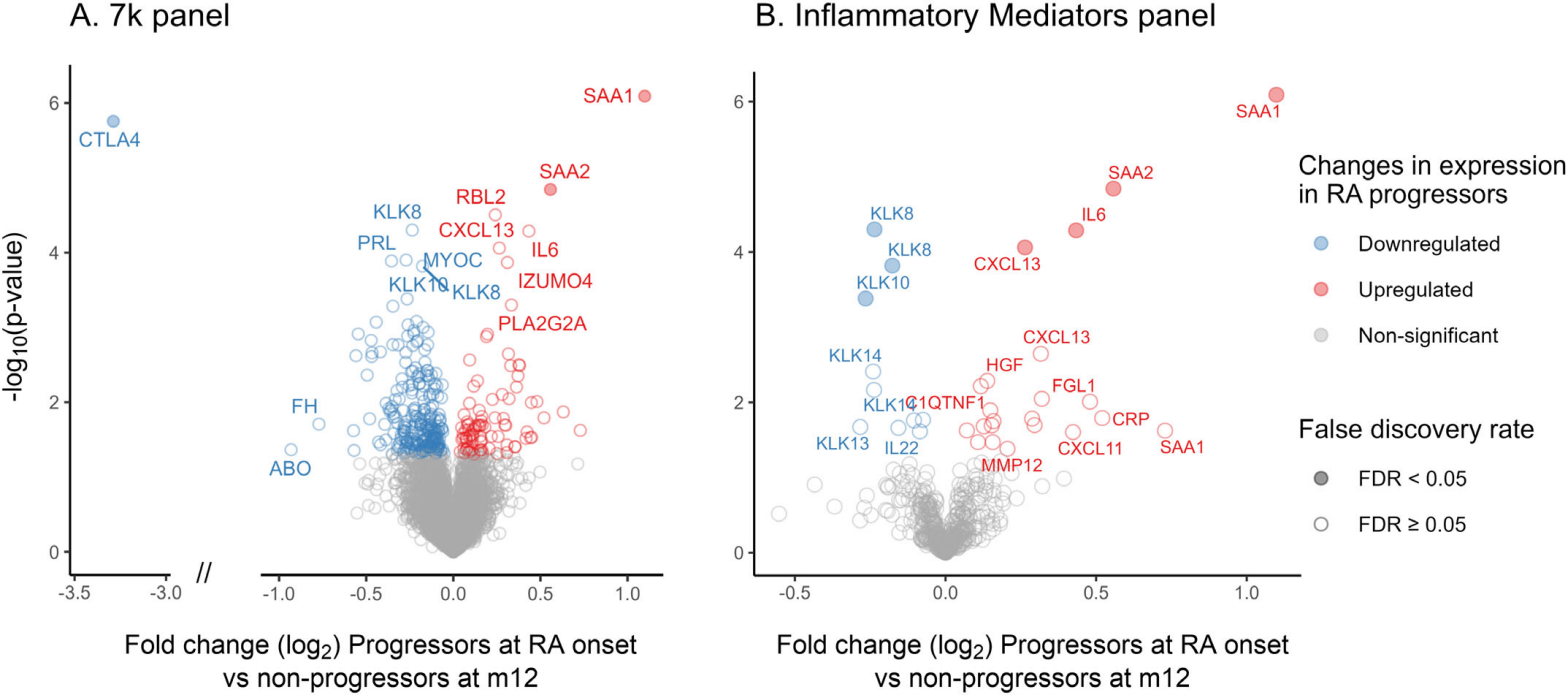
Serum protein expression comparing progressors of RA and nonprogressors. Volcano plot for differentially expressed proteins in progressors of RA at onset compared to nonprogressors at the end of treatment period regardless of arm selected from the (A) 7k panel and (B) Inflammatory Mediators panel. FDR, false discovery rate; m12, month 12/end of treatment; RA, rheumatoid arthritis.

Next, to identify proteomic differences that distinguish individuals who develop RA from those who remain disease‐free throughout the 24‐month study period, we compared the expression of serum proteins in progressors of RA at the time of disease onset with those of nonprogressors at the 12‐month time point (representing the end of treatment period), regardless of treatment arm (Figure 1C, step 2). We observed significant reductions in CTLA4 levels in those who progressed to RA (Figure 3A). Interestingly, the aptamer platform detects both endogenous CTLA4 and the CTLA4 subunits of the IgG fusion protein abatacept. Therefore, differential expression of CTLA4 that distinguishes between progressors of RA and nonprogressors likely reflects the levels of abatacept detected in the serum of participants randomized to the active arm and the absence of the study drug in serum of those in the placebo arm. In addition to down‐regulated CTLA4, we observed up‐regulated acute‐phase proteins SAA1 and SAA2 at disease onset in the 7k panel (Figure 3A). GO pathway analysis confirmed that inflammatory response was one of the most significant biologic processes associated with up‐regulated proteins (Supplementary Figure S8A). Up‐regulated IL‐6 JAK STAT3 signaling, interferon‐γ response, and inflammatory response GSEA pathways were associated with expressed proteins at RA onset compared to nonprogressors at month 12 regardless of allocated treatment (Supplementary Figure S8B). The Inflammatory Mediators panel revealed additional up‐regulated IL‐6 and CXCL13 and down‐regulated KLK8 and KLK10 (Figure 3B). Increased levels of SAA1, SAA2, IL‐6, and CXCL13 at RA onset visit are illustrated in Supplementary Figure S9A. In the Adaptive Immune Cell panel shown in Supplementary Figure S9B, we observed significant up‐regulated IL‐6 and down‐regulated CTLA4.

When we examined changes in placebo participants, we observed no differentially expressed proteins following FDR correction (Supplementary Figure S10A–C). Up‐regulated inflammatory response, TNF‐α signaling via NF‐κB, allograft rejection, and IL‐6 JAK STAT3 signaling GSEA pathways were associated with expressed proteins in placebo individuals at RA onset compared to nonprogressors at month 12 (Supplementary Figure S10D).

### Changes from baseline to RA onset

We also examined changes in protein levels from the at high‐risk state (baseline) to the time of definite RA, both overall and with respect to allocated treatment, to understand disease progression from baseline to RA diagnosis (Figure 1C, step 3). We found no differentially expressed proteins either in the 7k panel or the Inflammatory Mediators or Adaptive Immune Cell panels when analyzing all samples together, or in the placebo group (Supplementary Figures S11 and S12).

In the abatacept arm, we observed significantly down‐regulated CILP2 and up‐regulated RGS5 in the 7k panel (Supplementary Figure S13A). In the Inflammatory Mediators panel, we documented a more complex pattern with changes in expression at RA onset. IFNA10 and IL20RB were down‐regulated, and HGF, MMP9, IL10RB, and CCL18 were up‐regulated (Supplementary Figure S13B). CILP2 was the only protein down‐regulated in the panel of adaptive immune cells in abatacept participants at the time of RA onset (Supplementary Figure S13C).

### Treatment response signatures

Treatment effects on serum protein signatures were initially investigated by comparing serum protein levels at the end of treatment between participants randomized to abatacept and placebo, regardless of outcome (Figure 1C, step 4). Among 7,289 human protein‐binding aptamers, only up‐regulated CTLA4 showed differential expression (adjusted *P* value < 0.0001, 443‐FC) in abatacept participants. As noted earlier, CTLA4 reflected the detection of abatacept drug levels in the active treatment arm at the end of the treatment period. KEGG pathway analysis of down‐regulated proteins in the abatacept group showed cytokine‐cytokine receptor interaction (hsa04060), viral protein interaction with cytokine and cytokine receptor (hsa04061), and NF‐κB signaling pathway (hsa04064) (Supplementary Figure S14A). GO analysis indicated down‐regulated proteins were related to adaptive immune response (Supplementary Figure S14B). There was no significant KEGG pathway or GO biologic process associated with up‐regulated differentially expressed proteins in the abatacept group at month 12. In the Inflammatory Mediators panel, we observed down‐regulated CXCL13, CCL21, and LTA|LTB (Supplementary Figure S14C). In the Adaptive Immune Cell bespoke panel, we documented up‐regulated CTLA4 and downregulation of CXCL13 expression (Supplementary Figure S14D).

**Figure 4:**
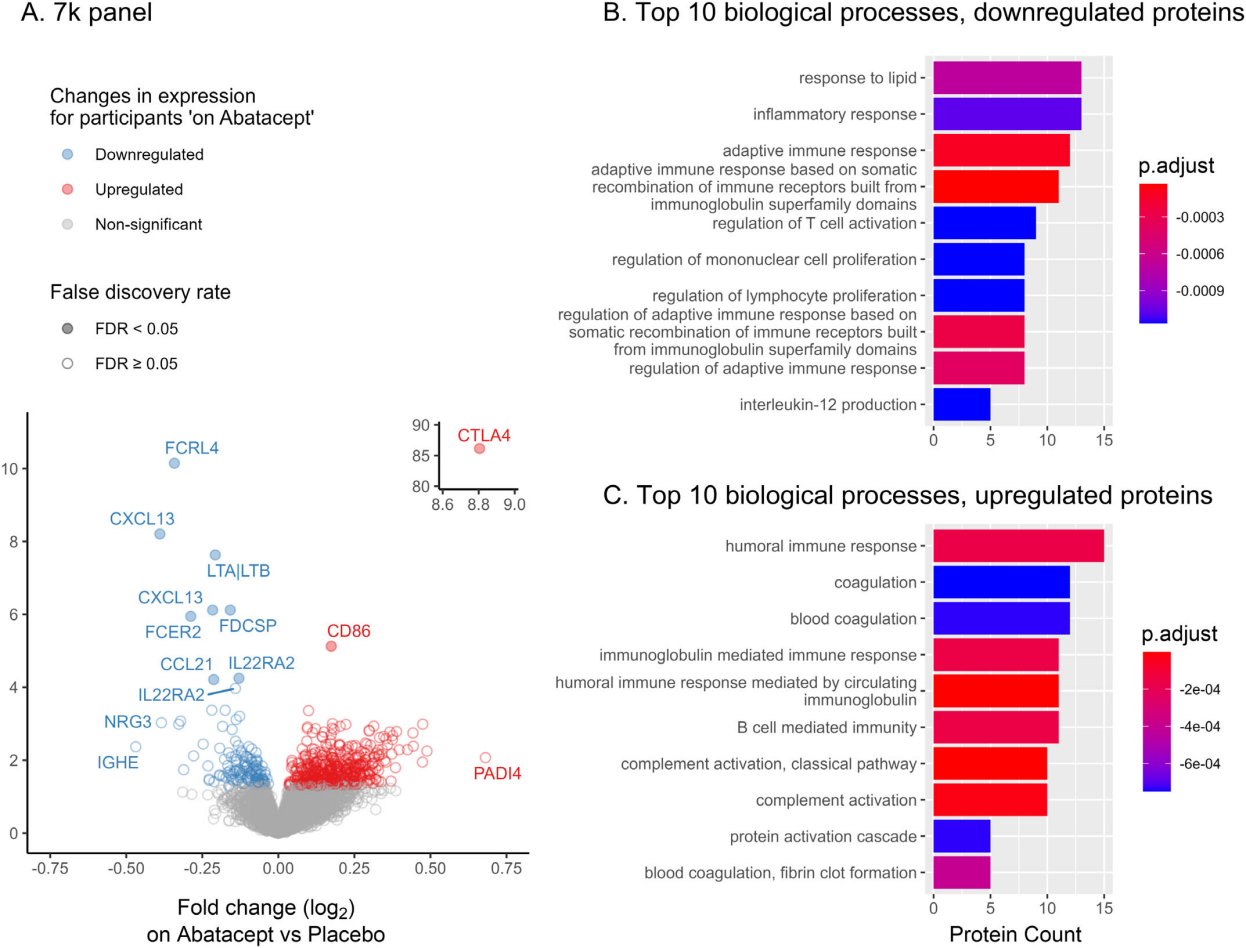
Effects of abatacept on serum protein profiles. (A) Volcano plot for differentially expressed proteins from the 7k panel in participants on abatacept. Top 10 Gene Ontology biologic processes for (B) down‐regulated and (C) up‐regulated proteins in participants treated with abatacept. FDR, false discovery rate; RA, rheumatoid arthritis.

To maximize statistical power, we performed complementary analyses comparing abatacept‐treated participants during the treatment period with placebo participants across the 24‐month study period, regardless of outcome (Figure 1C, step 5). This approach identified a broader treatment signature in the 7k panel. In participants treated with abatacept, we observed increased levels of CTLA4, as expected, but also the co‐stimulatory molecule CD86, as well as reduced levels of CXCL13, FCRL4, FCER2, CCL21, LTA|LTB, FDCSP, and IL22RA2 from the 7k panel (Figure 4A). The top three GO biologic processes associated with down‐regulated proteins were response to lipid, inflammatory response, and adaptive immune response (Figures 4B). Up‐regulated differentially expressed proteins were associated with humoral immune response and coagulation (Figures 4C). The top KEGG pathway analysis showed down‐regulated proteins were enriched in cytokine‐cytokine receptor interactions and viral protein interactions with cytokine‐cytokine receptor (Supplementary Figure S15A), whereas up‐regulated proteins were associated with systemic lupus erythematosus, ECM‐receptor interaction, and, the most significant being complement and coagulation cascades (Supplementary Figure S15B).

Consistent patterns were observed with our bespoke panels. In the Inflammatory Mediators panel, we observed additional down‐regulated IL12B and up‐regulated C4A|C4B and CXCL16 (Supplementary Figure S15C). The reliability of these findings is supported by Figures 4A and Supplementary Figure S15C, with two SOMAmer reagents targeting different epitopes on the same target protein CXCL13 showing a strong positive correlation (*r* = 0.86). In the Adaptive Immune Cell panel, we additionally observed reductions in TIGIT, TNFRSF1B, and IL12B (Supplementary Figure S15D).

To investigate whether changes observed represent clinically meaningful response indicators, we examined the differences in serum protein levels of CXCL13, SAA1, and IL‐6 at baseline and at months 12 to 15 in abatacept‐treated individuals. We did not observe significant differences in protein levels between abatacept progressors of RA and nonprogressors at the month 12 or 15 visit (Supplementary Figure S16), suggesting that the observed reductions during treatment and subsequent elevations after drug withdrawal appear to reflect a common pharmacologic effect of abatacept rather than a response‐specific mechanism.

Combining risk and response signatures (Figure 1C, steps 1 and 5) across all three panels, 17 proteins were observed to be both down‐regulated on abatacept and up‐regulated at RA onset (Supplementary Figure S17A), and 42 proteins were both up‐regulated on abatacept and down‐regulated at RA onset (Supplementary Figure S17B).

### Drug levels and outcome

**Figure 5:**
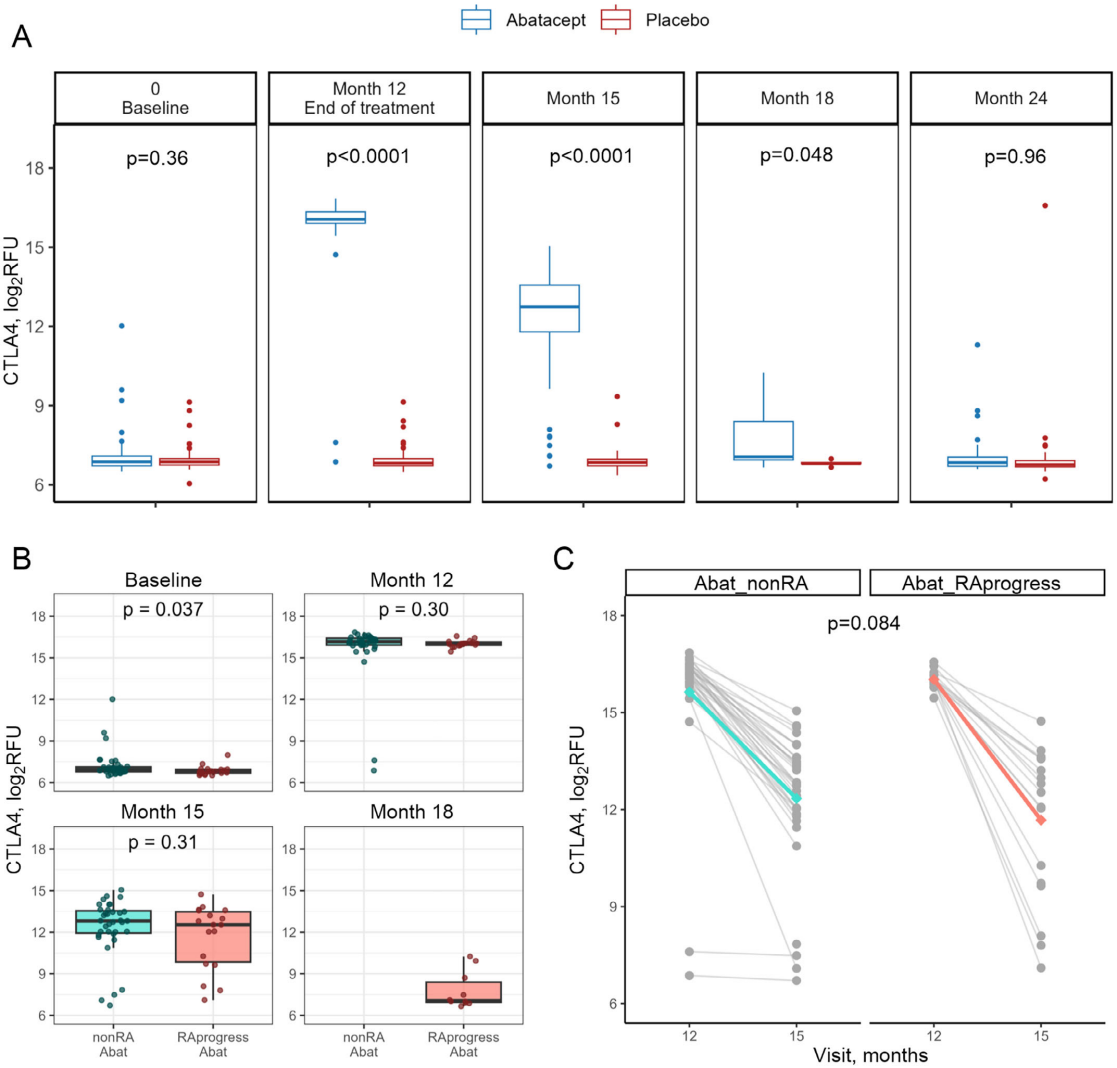
Longitudinal analysis of expression of serum CTLA4. (A) Longitudinal CTLA4 levels (log2 RFU) from baseline stratified by trial arm. (B) Longitudinal CTLA4 levels (log2 RFU) for abatacept participants stratified by RA outcome. (C) Difference in log2 fold change from month 12 to month 15 between progressors of RA and nonprogressors in the abatacept group. Highlighted lines indicate group means and connected dots of individual observations. Statistical significance was determined using Welch's *t*‐test with unequal variances. RA, rheumatoid arthritis; RFU, relative fluorescence units.

Monitoring log2‐transformed CTLA4 levels revealed similarity between two arms before starting treatment, with estimated means ± SD of 7.1 ± 0.9 log2 RFU for the abatacept and 7.0 ± 0.5 log2 RFU for the placebo group at baseline (Figure 5A). Significant differences in estimated means between arms were observed during the dosing period and up to month 18, six months after stopping the drug. CTLA4 levels peaked at month 12 for the abatacept group (mean ± SD: 15.8 ± 1.8 log2 RFU), gradually decreasing after stopping treatment, in keeping with drug washout (mean ± SD 12.1 ± 2.1 log2 RFU at month 15 and 7.8 ± 1.3 log2 RFU at month 18). A placebo outlier at month 24 was examined and robustness of results was confirmed by excluding this outlier in the sensitivity analysis. We compared baseline, month 12, and month 15 drug levels in abatacept participants stratified by outcome. Although abatacept participants who progressed to RA had lower baseline CTLA4 levels (log2 scale) than those who did not progress to RA, this disparity was no longer evident at the end of treatment or after treatment (Figure 5B), possibly reflecting the influence of outliers or random fluctuation rather than a persistent biologic difference. There was a nonsignificant trend toward greater reduction in CTLA4 levels between months 12 and 15 for those who progressed to RA (Figure 5C). Consistent with this, we observed no statistical difference in baseline CTLA4 levels for placebo participants stratified by RA outcome (Supplementary Figure S18).

## DISCUSSION

Traditionally, the identification of biomarker signatures associated with specific disease states has explored the expression of panels of proteins whose functions are largely known. Assays based on Luminex, O‐link or NuELISA platforms, for example, have measured tens to hundreds of cytokines, growth factors, matrix metalloproteinases, and chemokines, factors detectable in serum during active disease and linked to pathogenic processes. These platforms exploit highly sensitive tools to detect very low serum levels likely present in peripheral tissues at higher concentrations. More systematic, unbiased approaches have applied protein‐mass spectrometry to biologic fluids for which sensitivity of detection and quantification may be more limited. Here, we cast a wider net, opting for the aptamer platform to analyze >7,000 analytes that include well‐known factors associated with inflammatory responses, but also multiple cell‐derived proteins not conventionally considered secreted. These may reflect more subtle changes in cell activation or stress, as well as signs of metabolic dysfunction or reprogramming in which homeostatic mechanisms strive to balance energy consumption and preservation.

Taking advantage of the clinical trial setting and careful selection of samples across the spectrum of risk states and treatment responses, we made several key observations. First, we confirmed an acute‐phase response that evolves toward the time of RA onset, characterized by increased levels of IL‐6, SAA1, and SAA2. These are well established acute‐phase proteins that have been described previously, but in different clinical settings.^26^, ^27^, ^28^, ^29^ The chemokine CXCL13 emerges as another factor whose expression increases in serum as at‐risk individuals approach disease onset. This chemokine is particularly relevant to the at‐risk state, given that it is a product of T peripheral helper and follicular help cells and the promotion of T cell–dependent B cell responses and autoantibody production,^30^, ^31^ a critical signature of high‐risk individuals.^32^ We also identified other proteins associated with inflammatory states such as LILRA5, a member of the leukocyte immunoglobulin receptor family, associated with inflammatory cytokine production and promotion of antigen‐presenting cell function mostly in myeloid cells,^33^ and pappalysin, whose main function is to cleave IGF‐binding proteins and thereby regulate cell proliferation and survival, tissue modeling, and repair.^34^ It has been linked to RA in light of the fact that TNF‐α and IL‐1β induce its expression.^35^, ^36^

Second, we uncovered evidence that carbon metabolism, heme metabolism, and fatty acid metabolism are altered as individuals progress to disease, suggesting a metabolic shift as a consequence of changes in energy and biosynthetic demands. It is well known that metabolic reprogramming accompanies the immune response switch toward a proinflammatory profile.^37^, ^38^ Overall, proinflammatory responses are associated with increased glycolytic flux that fuels biosynthesis of key proliferative intermediates, for instance the pentose phosphate pathway,^39^ while mitochondrial OXPHOS capacity is reduced. We found significant downregulation in the tricarboxylic acid (TCA) components such as fumarate hydratase, a TCA enzyme whose downregulation might be expected to shift oxidative phosphorylation toward glycolysis, and cytoplasmic aconitate hydratase, which might indicate a reduction in itaconate production, and consequently, attenuation of anti‐inflammatory pathways.^40^ Other down‐modulated proteins linked to the TCA include alanine aminotransferase 1 and methylmalonyl‐CoA epimerase and suggest a broader metabolic suppression of mitochondrial function in both individuals in the placebo arm who progress within the first 12 months and individuals who progress upon stopping abatacept, indicating poor resolution of inflammatory responses. Importantly these are signatures that precede the inset of clinically detectable joint disease.^41^

A priority of our study was to address changes in protein signatures arising as a consequence of 12 months of abatacept treatment (Figure 4). Five of the most significant proteins down‐regulated by drug—CXCL13, LTA/LTB, FCRL4, FDCSP, and FCER2—point to a common immune pathway associated with B cell recruitment, lymphoid tissue organization, germinal center formation, activation of follicular dendritic cells and tissue‐resident B cells, and B cell signaling.^34^ In the absence of detectable joint inflammation in the at‐risk trial population,^9^ one assumption is that these processes may be active in secondary lymphoid organs during the at‐risk phase.^42^ Another possibility is that early immune interactions may reside in peripheral tissues such as the lung or gut, which would be in keeping with changes in IL22RA2 production which plays a role in mucosal immunity and epithelial repair.^43^

Abatacept is a CTLA4‐Ig fusion protein that modulates the CD28‐CD80/CD86 co‐stimulatory pathway, leading to inhibition of T cell activation^44^, ^45^ and attenuation of proliferation and proinflammatory cytokine production, which are key processes in the initiation of autoimmune responses that lead to RA. This interaction may also trigger compensatory upregulation of CD86 on antigen‐presenting cells. Observed upregulation of CTLA4 and CD86 in participants at risk of RA receiving abatacept therapy likely reflects abatacept's mechanism of action and suggests that regulation of co‐stimulatory signaling, including the shedding of surface receptors into extracellular compartments, may underpin immune modulation associated with abatacept therapy during this preclinical phase. More broadly, these findings highlight the therapeutic potential of targeting co‐stimulatory pathways both for the treatment of established RA and for prevention of disease development in at‐risk individuals.

A key limitation of our study is the relatively small sample sizes within subgroups of progressors of RA, nonprogressors, or treatment groups. This limitation may have reduced statistical power, increasing the likelihood of Type II errors, and the ability to detect true differences between groups. Therefore, nonsignificant results should be interpreted with caution. Our analysis of many thousands of proteins and application of a correction for FDR also means we may miss modulation of key proteins as either markers of risk or of drug response. To enhance discovery, FDR correction was applied separately within each subpanel, although overlap among subpanels means that the overall FDR control cannot be guaranteed. Second, future research with larger and more balanced subgroup representation is required to validate these results in independent cohorts and enhance generalizability. Another limitation of this study is that the results may reflect, in part, the pharmacological effect of abatacept rather than disease or response‐associated changes, a good example being the signature including CXCL13 suggestive of T follicular helper/T peripheral helper cell activation, subsets known to be targeted by abatacept.^46^ Future work should focus on longitudinal profiling including pharmacokinetic data and appropriate control groups. However, strengths of the study include robust study outcomes and analysis of samples across the duration of the study, as well as the opportunity to study the impact of an intervention and an unbiased identification of an adaptive immune cell signature that is modified when at‐risk individuals are treated with abatacept and when rates of progression to RA are at their lowest.

In conclusion, our data support approaches for studying cellular and molecular features of the at‐risk state and for applying assays for just a small number of proteins over periods of time to refine this state. This could focus on acute‐phase proteins such as IL‐6, SAA1, and SAA2 and the chemokine CXCL13 as a biomarker of disease promoting adaptive immune responses. Protein signatures identified in this study have potential utility in clinical risk stratification and monitoring of treatment response, especially those implicated in the acute‐phase response, and the T‐B cell cluster. In future studies, longitudinal evaluation of these markers in at‐risk individuals will help identify individuals at higher risk of developing RA and guide early therapeutic interventions to prevent or delay the disease onset. Further work is also required to explore in more depth associations between proteomic changes and clinical outcomes and to better understand the metabolic and mitochondrial dysfunction, which appears to precede the onset of clinically apparent disease.

## AUTHOR CONTRIBUTIONS

All authors contributed to at least one of the following manuscript preparation roles: conceptualization AND/OR methodology, software, investigation, formal analysis, data curation, visualization, and validation AND drafting or reviewing/editing the final draft. As corresponding author, M Jasenecova confirms that all authors have provided the final approval of the version to be published and takes responsibility for the affirmations regarding article submission (eg, not under consideration by another journal), the integrity of the data presented, and the statements regarding compliance with institutional review board/Declaration of Helsinki requirements.

## ROLE OF THE STUDY FUNDER

Bristol Myers Squibb had no role in data collection, data analysis, data interpretation, or writing of the report. This proteomic study was supported by the APIPPRA study grant from Bristol Myers Squibb (BMS). APIPPRA was an investigator‐led study funded by BMS and sponsored by King's College London, Guy's and St Thomas' NHS Foundation Trust, and Leiden University Medical Centre. BMS had no role as study sponsor.

## Supporting information


**Disclosure Form**:

**Data S1.** Supporting Information.
