# Supplementary material for: Serum Proteomic Signatures of Rheumatoid Arthritis Risk and Response: Analysis of a Rheumatoid Arthritis Interception Trial
Source: Arthritis Rheumatol. 2026 Feb 17;78(7):1403–13. doi: 10.1002/art.70082 (PMC13313106; doi:10.1002/art.70082)
Supplement: Supplementary file 2 — Data S1. Supporting Information. [file ART-78-1403-s002.docx]

Supplementary

Table of Contents

[7k SomaScan® assay and bespoke panels 2](#_Toc216710880)

[Quality control 2](#_Toc216710881)

[Table S1. Differential expression comparing RA onset and 6-24 months pre-RA 3](#_Toc216710882)

[Figure S1. Gene Set Enrichment Analysis (GSEA) pathways for differentially expressed proteins at RA onset compared to 6-24 months pre-RA from the 7k panel 5](#_Toc216710883)

[Figure S2. Chord diagram for top correlated differentially expressed proteins at RA onset compared to 6-24 months pre-RA 6](#_Toc216710884)

[Figure S3. Differentially expressed proteins at RA onset compared to 6-24 months pre-RA 7](#_Toc216710885)

[Figure S4. Differentially expressed proteins at RA onset compared to 3-6 months pre-RA 8](#_Toc216710886)

[Figure S5. GSEA pathways for differentially expressed proteins at RA onset compared to 3-6 months pre-RA from the 7k panel 9](#_Toc216710887)

[Figure S6. Differentially expressed proteins at RA onset compared to up to 3 months pre-RA 10](#_Toc216710888)

[Figure S7. GSEA pathways for differentially expressed proteins at RA onset compared to 0-3 months pre-RA from the 7k panel 11](#_Toc216710889)

[Figure S9. Differentially expressed proteins from the Inflammatory Mediators and Adaptive Immune Cell panel in RA progressors at RA onset compared to non-progressors at the end of treatment regardless of treatment arm 13](#_Toc216710890)

[Figure S10. Pathway analysis for differentially expressed proteins in serum from RA progressors compared to non-progressors in the placebo arm 15](#_Toc216710891)

[Figure S11. Changes from baseline to RA onset regardless of trial arm 16](#_Toc216710892)

[Figure S12. Changes from baseline to RA onset for placebo arm 17](#_Toc216710893)

[Figure S13. Changes from baseline to RA onset for abatacept arm 18](#_Toc216710894)

[Figure S14. Pathway analysis for differentially expressed proteins in serum from participants randomised to abatacept compared to placebo at the end of treatment at month 12 regardless of outcome 19](#_Toc216710895)

[Figure S15. Pathway analysis for differentially expressed proteins in serum from participants ‘on abatacept’ vs placebo 20](#_Toc216710896)

[Figure S16. Longitudinal analysis of expression of serum CXCL13, SAA1, and IL-6 in abatacept participants stratified by progression outcome 22](#_Toc216710897)

[Figure S17. Venn diagram with intersection table for differentially expressed proteins associated with risk and response 24](#_Toc216710898)

[Figure S18. Baseline CTLA4 (log2 scale) expression in placebo group stratified by progression outcome 25](#_Toc216710899)

[REFERENCES 26](#_Toc216710900)

[Statistical Analysis Plan 27](#_Toc216710901)

7k SomaScan® assay and bespoke panels

Participants’ serum was separated in the study contracted laboratories and transferred to NIHR National Biosample Centre (UK Biocentre) in Milton Keynes for archiving. Selected samples were processed and then analysed in SomaLogic laboratories (SomaLogic, Inc., Boulder, CO, USA), as described^1^. Protein levels were expressed as arbitrary relative fluorescence units (RFU), considered directly proportional to the amount of the target epitope present in the sample. Here, the SomaScan® assay v4.1^2^ was used, comprising approximately 7k SOMAmer (Slow Off-Rate Modified Aptamer) human protein-binding reagents. To understand how protein relevant to specific pathways changed, we defined two pre-specified bespoke subpanels, comprised of 479 inflammatory mediators and 373 associated with adaptive immune responses (hereafter, “Inflammatory Mediators” and “Adaptive Immune Cell” panels) (Figure 1B). SomaScan® output files included metadata and quality statements with the key quality attributes. A series of normalisation and hybridization steps were applied to raw data to account for intra- and inter-plate variability of buffer, calibrator, quality control, and experimental samples, as previously described^3^. Data from 67 additional quality control samples for buffer (n=19), calibrator (n=30), as well as samples from 18 healthy donors were removed prior to analysis. The assay comprised, in total, 7,596 SOMAmer aptamers - 26 non-human, 20 spuriomer, 12 hybridization controls for elution, ten non-biotin, four non-cleavable, 233 aptamers detecting mouse proteins, one HIV1, one HIV2 and 7,289 SOMAmer reagents targeting annotated human proteins. In the 7K assay, the 7,289 SOMAmers were linked to 6,384 unique UniProt IDs, 6,378 unique Entrez gene IDs, and 6,366 unique Entrez gene symbols determined from the data file annotations.

Quality control

A total of 99% (436/440) of APIPPRA samples passed quality control criteria, while 1% (4/440) of samples labelled as placebo participant at baseline, placebo month 12 RA onset visit, placebo month 12, abatacept at month 15 were flagged across all SOMAmer reagents during quality control assessment. Four potential outliers identified in the dataset were retained in main analyses. Out of 7,289 SOMAmer reagents targeting human proteins, 10% were flagged across all APIPPRA visits and retained for analyses as recommended by the manufacturer’s quality statement.

Table S1. Differential expression comparing RA onset and 6-24 months pre-RA

| **Target** | **Target Full Name** | **UniProt** | **log2_FC** | **FC** | **pval** | **fdr** | **Changes in expression at**  **RA onset** |
| --- | --- | --- | --- | --- | --- | --- | --- |
| FH | Fumarate hydratase, mitochondrial | P07954 | -1 | 0.5 | <0.0001 | 0.026 | down |
| APOB | Apolipoprotein B | P04114 | -0.91 | 0.53 | <0.0001 | 0.033 | down |
| CDYL2 | Chromodomain Y-like protein 2 | Q8N8U2 | -0.86 | 0.55 | <0.0001 | 0.026 | down |
| NIF3L1 | NIF3-like protein 1 | Q9GZT8 | -0.83 | 0.56 | <0.0001 | 0.026 | down |
| LTA4H | Leukotriene A-4 hydrolase | P09960 | -0.75 | 0.6 | 0.00026 | 0.044 | down |
| GDI1 | Rab GDP dissociation inhibitor alpha | P31150 | -0.66 | 0.63 | <0.0001 | 0.026 | down |
| APOC3 | Apolipoprotein C-III | P02656 | -0.66 | 0.63 | 0.00016 | 0.04 | down |
| UNG | Uracil-DNA glycosylase | P13051 | -0.59 | 0.66 | 0.00020 | 0.044 | down |
| OASL | 2'-5'-oligoadenylate synthetase like protein | Q15646 | -0.55 | 0.68 | <0.0001 | 0.0041 | down |
| ACO1 | Cytoplasmic aconitate hydratase | P21399 | -0.54 | 0.69 | <0.0001 | 0.031 | down |
| GBP2 | Guanylate-binding protein 2 | P32456 | -0.53 | 0.69 | 0.00036 | 0.044 | down |
| ALAD | Delta-aminolevulinic acid dehydratase | P13716 | -0.49 | 0.71 | 0.00025 | 0.044 | down |
| SNAP23 | Synaptosomal-associated protein 23 | O00161 | -0.48 | 0.72 | <0.0001 | 0.026 | down |
| PCLAF | PCNA-associated factor | Q15004 | -0.48 | 0.72 | <0.0001 | 0.033 | down |
| CAPZA1 | F-actin-capping protein subunit alpha-1 | P52907 | -0.48 | 0.72 | 0.00051 | 0.047 | down |
| CCT7 | T-complex protein 1 subunit eta | Q99832 | -0.47 | 0.72 | 0.00030 | 0.044 | down |
| A2M | Alpha-2-macroglobulin | P01023 | -0.46 | 0.73 | 0.00032 | 0.044 | down |
| MACROD2 | O-acetyl-ADP-ribose deacetylase MACROD2 | A1Z1Q3 | -0.45 | 0.73 | 0.00044 | 0.046 | down |
| XPNPEP1 | Xaa-Pro aminopeptidase 1 | Q9NQW7 | -0.45 | 0.73 | 0.00050 | 0.047 | down |
| RNPEP | Aminopeptidase B | Q9H4A4 | -0.4 | 0.76 | 0.00042 | 0.046 | down |
| TKFC | Dihydroxyacetone kinase | Q3LXA3 | -0.39 | 0.76 | 0.00014 | 0.038 | down |
| PEA15 | Astrocytic phosphoprotein PEA-15 | Q15121 | -0.38 | 0.77 | 0.00012 | 0.038 | down |
| ENO2 | Gamma-enolase | P09104 | -0.38 | 0.77 | 0.00027 | 0.044 | down |
| TRMT61B | tRNA (adenine(58)-N(1))-methyltransferase, mitochondrial | Q9BVS5 | -0.38 | 0.77 | 0.00019 | 0.044 | down |
| UROD | Uroporphyrinogen decarboxylase | P06132 | -0.37 | 0.77 | 0.00046 | 0.047 | down |
| CLTA | Clathrin light chain A | P09496 | -0.36 | 0.78 | 0.00025 | 0.044 | down |
| NUBP2 | Cytosolic Fe-S cluster assembly factor NUBP2 | Q9Y5Y2 | -0.35 | 0.78 | 0.00032 | 0.044 | down |
| PREP | Prolyl endopeptidase | P48147 | -0.34 | 0.79 | 0.00024 | 0.044 | down |
| USP5 | Ubiquitin carboxyl-terminal hydrolase 5 | P45974 | -0.34 | 0.79 | <0.0001 | 0.033 | down |
| SLC5A8 | Sodium-coupled monocarboxylate transporter 1 | Q8N695 | -0.33 | 0.79 | <0.0001 | 0.0041 | down |
| SCFD1 | Sec1 family domain-containing protein 1 | Q8WVM8 | -0.32 | 0.8 | <0.0001 | 0.033 | down |
| UBA3 | NEDD8-activating enzyme E1 catalytic subunit | Q8TBC4 | -0.32 | 0.8 | 0.00044 | 0.046 | down |
| CRYBA2 | Beta-crystallin A2 | P53672 | -0.3 | 0.81 | 0.00028 | 0.044 | down |
| UBE4A | Ubiquitin conjugation factor E4 A | Q14139 | -0.29 | 0.82 | 0.00044 | 0.046 | down |
| RPP25 | Ribonuclease P protein subunit p25 | Q9BUL9 | -0.28 | 0.82 | <0.0001 | 0.033 | down |
| SIRT2 | NAD-dependent protein deacetylase sirtuin-2 | Q8IXJ6 | -0.27 | 0.83 | 0.00015 | 0.038 | down |
| THOP1 | Thimet oligopeptidase | P52888 | -0.27 | 0.83 | <0.0001 | 0.033 | down |
| ANKRA2 | Ankyrin repeat family A protein 2 | Q9H9E1 | -0.27 | 0.83 | <0.0001 | 0.026 | down |
| KCNMB3 | Calcium-activated potassium channel subunit beta-3 | Q9NPA1 | -0.26 | 0.83 | 0.0005 | 0.047 | down |
| LDLRAD4 | Low-density lipoprotein receptor class A domain-containing protein 4 | O15165 | -0.24 | 0.85 | 0.00011 | 0.036 | down |
| MCEE | Methylmalonyl-CoA epimerase, mitochondrial | Q96PE7 | -0.22 | 0.86 | 0.00023 | 0.044 | down |
| CHMP6 | Charged multivesicular body protein 6 | Q96FZ7 | -0.21 | 0.86 | 0.00037 | 0.044 | down |
| EDIL3 | EGF-like repeat and discoidin I-like domain-containing protein 3 | O43854 | -0.21 | 0.87 | 0.00030 | 0.044 | down |
| GSTT1 | Glutathione S-transferase theta-1 | P30711 | -0.2 | 0.87 | 0.00038 | 0.044 | down |
| IL20RB | Interleukin-20 receptor subunit beta | Q6UXL0 | -0.2 | 0.87 | 0.00014 | 0.038 | down |
| NPY | Neuropeptide Y | P01303 | -0.19 | 0.88 | 0.00013 | 0.038 | down |
| ITM2B | Integral membrane protein 2B | Q9Y287 | -0.19 | 0.88 | 0.00046 | 0.047 | down |
| GPT | Alanine aminotransferase 1 | P24298 | -0.18 | 0.88 | <0.0001 | 0.033 | down |
| ROBO4 | Roundabout homolog 4 | Q8WZ75 | -0.17 | 0.89 | 0.00028 | 0.044 | down |
| PCSK1N | ProSAAS | Q9UHG2 | -0.17 | 0.89 | <0.0001 | 0.026 | down |
| BMP4 | Bone morphogenetic protein 4 | P12644 | -0.16 | 0.89 | 0.00030 | 0.044 | down |
| HPGDS | Hematopoietic prostaglandin D synthase | O60760 | -0.15 | 0.9 | 0.00031 | 0.044 | down |
| CA10 | Carbonic anhydrase-related protein 10 | Q9NS85 | -0.14 | 0.91 | <0.0001 | 0.026 | down |
| ETNK2 | Ethanolamine kinase 2 | Q9NVF9 | -0.14 | 0.91 | 0.00041 | 0.046 | down |
| PKN1 | Serine/threonine-protein kinase N1 | Q16512 | -0.13 | 0.91 | 0.00033 | 0.044 | down |
| PDIA3 | Protein disulfide-isomerase A3 | P30101 | -0.13 | 0.91 | 0.00053 | 0.049 | down |
| SULT6B1 | Sulfotransferase 6B1 | Q6IMI4 | -0.13 | 0.91 | 0.00043 | 0.046 | down |
| ATL3 | Atlastin-3 | Q6DD88 | -0.12 | 0.92 | <0.0001 | 0.026 | down |
| ZNF34 | Zinc finger protein 34 | Q8IZ26 | -0.12 | 0.92 | 0.00034 | 0.044 | down |
| CYB5R3 | NADH-cytochrome b5 reductase 3 | P00387 | -0.12 | 0.92 | 0.00021 | 0.044 | down |
| CCDC25 | Coiled-coil domain-containing protein 25 | Q86WR0 | -0.12 | 0.92 | <0.0001 | 0.033 | down |
| ITGA11\|ITGB1 | Integrin a11b1 | Q9UKX5\|P05556 | -0.11 | 0.93 | <0.0001 | 0.026 | down |
| CUL3 | Cullin-3 | Q13618 | -0.1 | 0.93 | 0.00033 | 0.044 | down |
| PHOSPHO1 | Phosphoethanolamine/phosphocholine phosphatase | Q8TCT1 | -0.1 | 0.93 | 0.00023 | 0.044 | down |
| RCN3 | Reticulocalbin-3 | Q96D15 | -0.09 | 0.94 | 0.00044 | 0.046 | down |
| CETN1 | Centrin-1 | Q12798 | -0.09 | 0.94 | 0.00043 | 0.046 | down |
| EFHD1 | EF-hand domain-containing protein D1 | Q9BUP0 | -0.09 | 0.94 | 0.00028 | 0.044 | down |
| EGF | Epidermal growth factor: Cytoplasmic domain | P01133 | -0.07 | 0.95 | 0.00033 | 0.044 | down |
| PCDHB1 | Protocadherin beta-1 | Q9Y5F3 | 0.05 | 1.04 | 0.00052 | 0.048 | up |
| GALNT11 | Polypeptide N-acetylgalactosaminyltransferase 11 | Q8NCW6 | 0.06 | 1.04 | 0.00020 | 0.044 | up |
| GRB7 | Growth factor receptor-bound protein 7 | Q14451 | 0.06 | 1.04 | 0.00036 | 0.044 | up |
| METTL1 | tRNA (guanine-N(7)-)-methyltransferase | Q9UBP6 | 0.07 | 1.05 | 0.00035 | 0.044 | up |
| CDK16 | Cyclin-dependent kinase 16 | Q00536 | 0.07 | 1.05 | 0.00023 | 0.044 | up |
| COMT | Catechol O-methyltransferase | P21964 | 0.07 | 1.05 | 0.00049 | 0.047 | up |
| HPSE | Heparanase | Q9Y251 | 0.08 | 1.06 | 0.00014 | 0.038 | up |
| LILRA5 | Leukocyte immunoglobulin-like receptor subfamily A member 5 | A6NI73 | 0.09 | 1.07 | 0.00017 | 0.04 | up |
| CRYZL1 | Quinone oxidoreductase-like protein 1 | O95825 | 0.17 | 1.12 | 0.00048 | 0.047 | up |
| PAPPA | Pappalysin-1 | Q13219 | 0.26 | 1.19 | 0.00026 | 0.044 | up |
| OXCT1 | Succinyl-CoA:3-ketoacid-coenzyme A transferase 1, mitochondrial | P55809 | 0.3 | 1.23 | 0.00047 | 0.047 | up |
| IL6 | Interleukin-6 | P05231 | 0.33 | 1.26 | 0.00034 | 0.044 | up |

FC=Fold Change; pval= Unadjusted p-value; fdr= False Discovery Rate/Adjusted p-value; RA=Rheumatoid Arthritis.


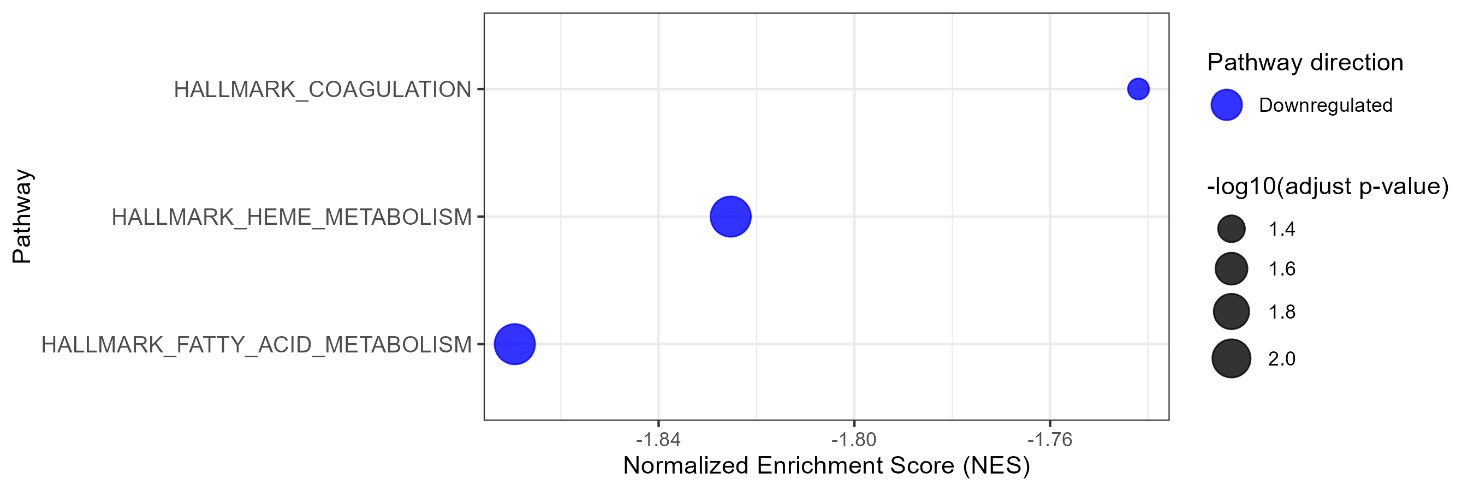


| ID | core_enrichment |
| --- | --- |
| HALLMARK_COAGULATION | HRG/ITGB3/SIRT2/PREP/A2M/APOC3/LTA4H/C3 |
| HALLMARK_HEME_METABOLISM | HAGH/HEBP1/TCEA1/CLIC2/UBAC1/PSMD9/BLVRB/HMBS/UROD/EPB41/SNCA/ALAD/C3 |
| HALLMARK_FATTY_ACID_METABOLISM | MCEE/GPD1/PRDX6/YWHAH/CBR1/ECHS1/LDHA/ UROD/ENO2/ALAD/MIF/FH |

Figure S1. Gene Set Enrichment Analysis (GSEA) pathways for differentially expressed proteins at RA onset compared to 6-24 months pre-RA from the 7k panel


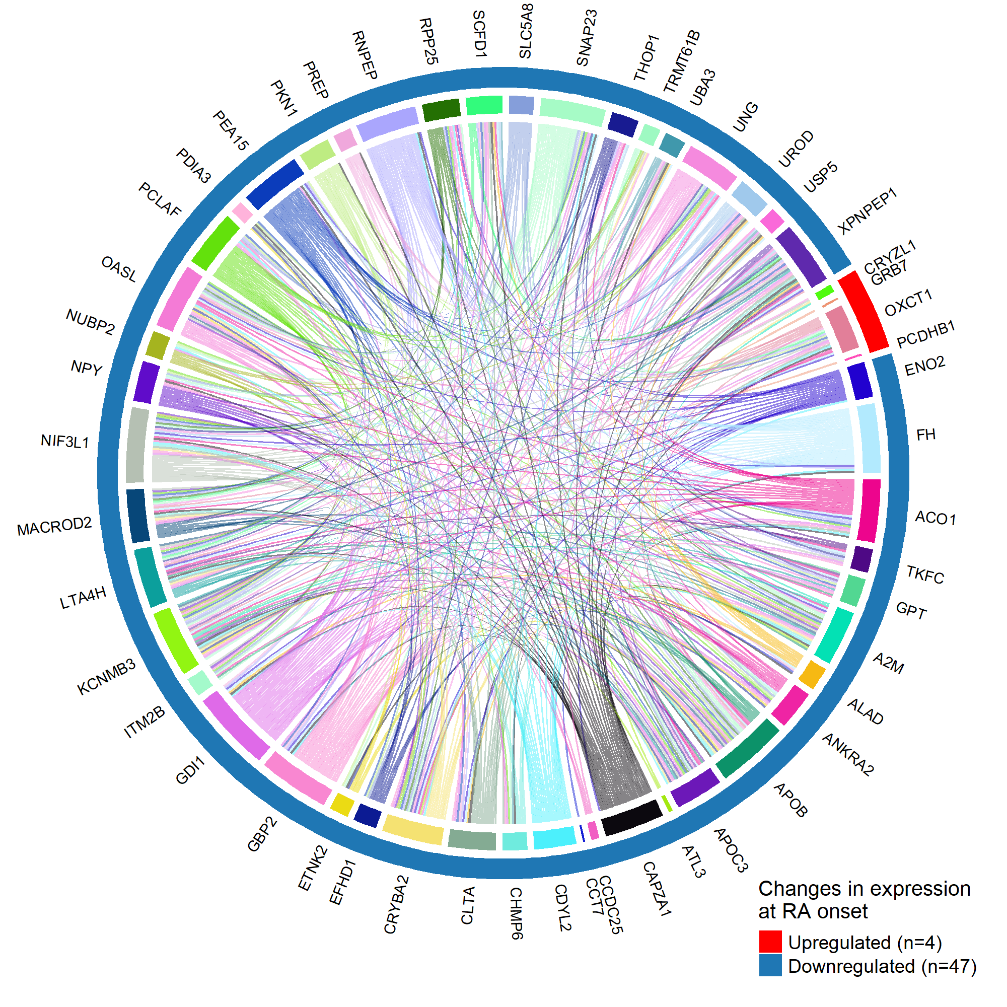


Figure S2. Chord diagram for top correlated differentially expressed proteins at RA onset compared to 6-24 months pre-RA

Absolute value of the Spearman’s rank correlation coefficient for illustrated proteins was over 0.7 and implies strong monotonic relationship between 2 proteins across all study visits (consistently increasing or decreasing, but not necessarily at a constant rate). Longer sector arcs in the chord diagram correspond to proteins with more connections, highlighting their potential importance in the biological network. Protein GDI1 presented strong associations with the most (n=37) other highly correlated displayed proteins, followed by NIF3L1 (n=35), GBP2, APOB (n=33), and FH (n=32). The strongest negative associations were shown between downregulated OASL, GDI1, PKN1 and upregulated OXCT1 (coefficient -0.75). The strongest positive associations were observed between downregulated proteins: OASL and SLC5A8 (coefficient 0.94), OASL and ANKRA2 (coefficient 0.91), and APOB and SNAP23 (coefficient 0.91). Downregulated ENO2/FH/ACO1/TKFC/GPT/MCEE at RA onset were associated with carbon metabolism pathway and except for MCEE, proteins ENO2, FH, ACO1, TKFC, GPT were shown in this chord diagram. Protein annotation can be found in Table S1.


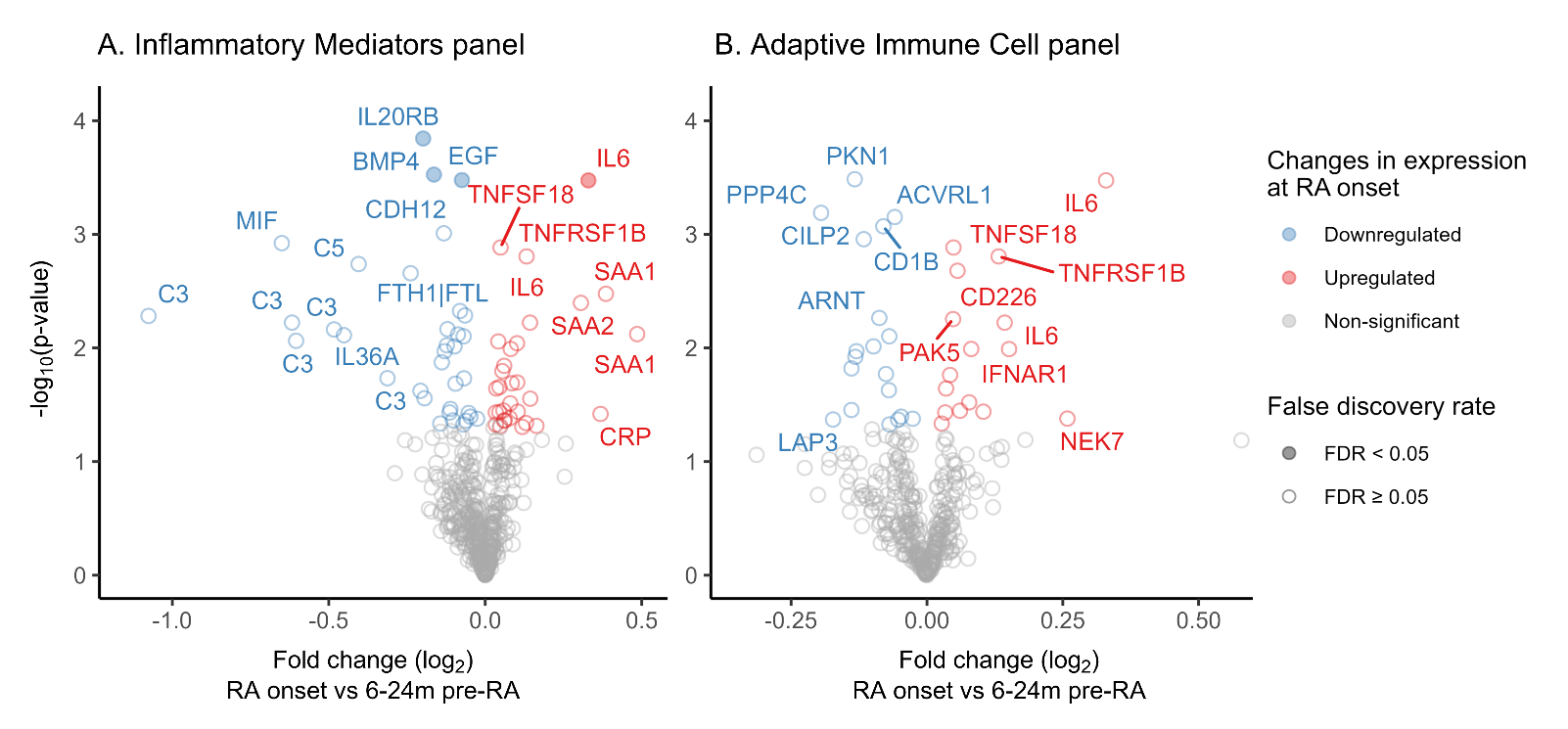


Figure S3. Differentially expressed proteins at RA onset compared to 6-24 months pre-RA

1. RA onset vs 6-24 pre-RA in Inflammatory Mediators panel N=479 with downregulated differentially expressed IL20RB, BMP4 and EGF and upregulated differentially expressed IL-6
2. RA onset vs 6-24 pre-RA in Adaptive Immune Cell panel N=373 with no differentially expressed proteins following Benjamini-Hochberg correction

BMP4=Bone morphogenetic protein 4; EGF=Epidermal growth factor; FDR=False Discovery Rate; IL-6=Interleukin-6; IL20RB=Interleukin-20 receptor subunit beta; RA=Rheumatoid Arthritis; 6-24m=6-24 months.


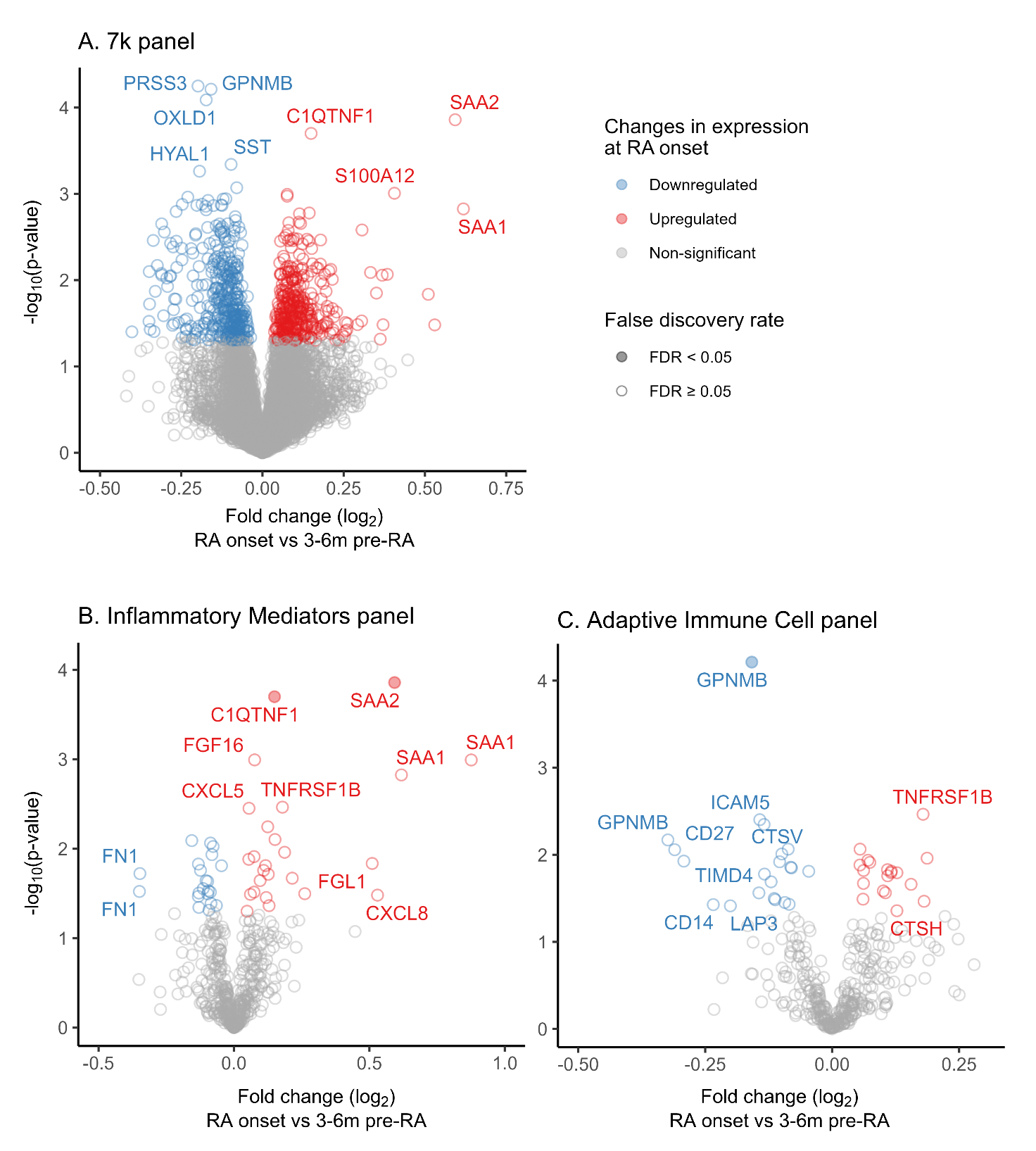


Figure S4. Differentially expressed proteins at RA onset compared to 3-6 months pre-RA

1. RA onset vs 3-6 months pre-RA in the 7k panel with no differentially expressed proteins following Benjamini-Hochberg correction
2. RA onset vs 3-6 months pre-RA in Inflammatory Mediators panel N=479 with upregulated differentially expressed C1QTNF1 and SAA2
3. RA onset vs 3-6 months pre-RA in Adaptive Immune Cell panel N=373 with downregulated differentially expressed GPNMB

C1QTNF1=Complement C1q tumor necrosis factor-related protein 1; FDR=False Discovery Rate; SAA2=Serum amyloid A-2 protein; GPNMB=Transmembrane glycoprotein NMB; 3-6m=3-6 months.


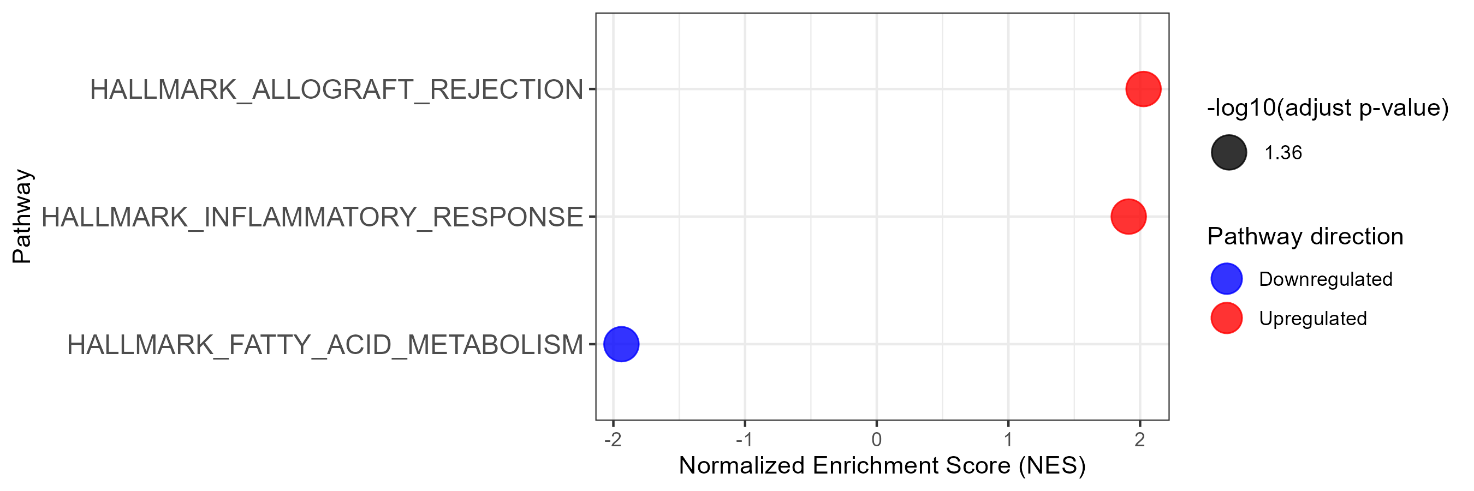


| ID | core_enrichment |
| --- | --- |
| HALLMARK_ALLOGRAFT_REJECTION | SPI1/CCL7/HCLS1/ELANE/CXCL13 |
| HALLMARK_INFLAMMATORY_RESPONSE | CXCL8/OLR1/CCL7/IFNAR1/TNFRSF1B/MARCO/RGS1 |
| HALLMARK_FATTY_ACID_METABOLISM | HSD17B10/CA6/ME1/UBE2L6/ALDOA/ECHS1 |

Figure S5. GSEA pathways for differentially expressed proteins at RA onset compared to 3-6 months pre-RA from the 7k panel


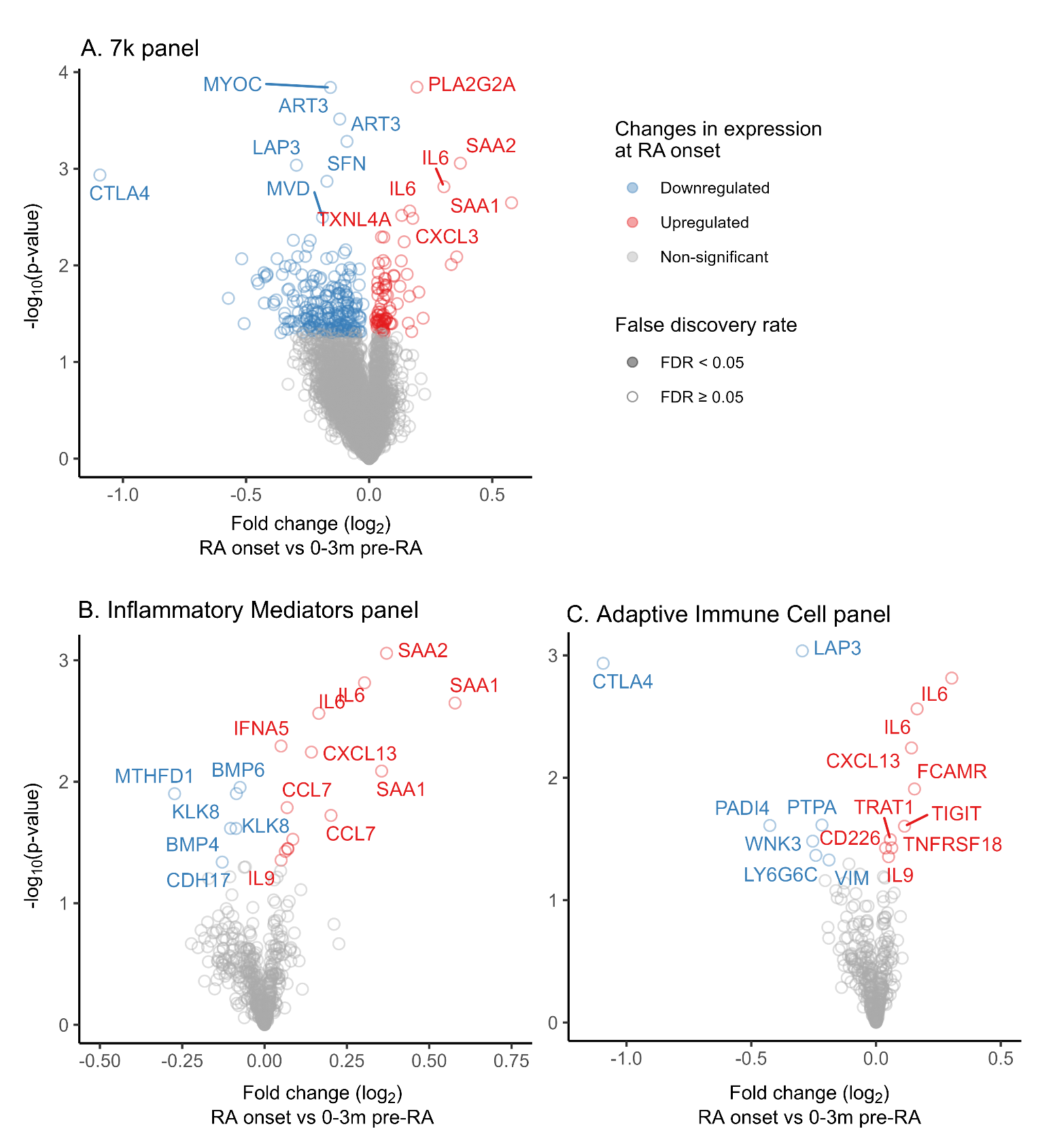
Figure S6. Differentially expressed proteins at RA onset compared to up to 3 months pre-RA

1. RA onset vs up to 3 months pre-RA in 7k panel with no differentially expressed proteins following Benjamini-Hochberg correction. There were 317 (234 downregulated and 83 upregulated) differentially expressed proteins in the sensitivity analysis (unadjusted p-value < 0.05).
2. RA onset vs up to 3 months pre-RA in Inflammatory Mediators panel N=479 with no differentially expressed proteins following Benjamini-Hochberg correction
3. RA onset vs up to 3 months pre-RA in Adaptive Immune Cell panel N=373 with no differentially expressed proteins following Benjamini-Hochberg correction

FDR=False Discovery Rate; RA=Rheumatoid Arthritis; 0-3m=0-3 months


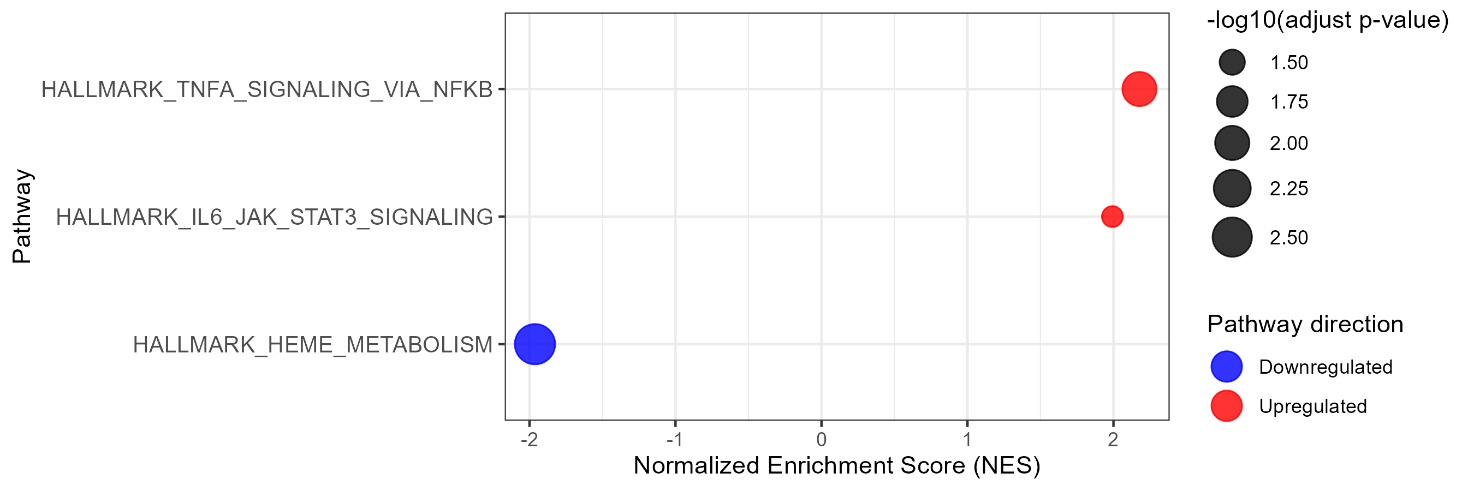


| ID | core_enrichment |
| --- | --- |
| HALLMARK_TNFA_SIGNALING_VIA_NFKB | IL6/CXCL3/VEGFA/BIRC2/BCL6/SAT1/ZBTB10 |
| HALLMARK_IL6_JAK_STAT3_SIGNALING | IL6/PLA2G2A/CXCL3/CXCL13/CCL7 |
| HALLMARK_HEME_METABOLISM | UBAC1/UROD/USP15/GCLM/CLIC2/TCEA1/HAGH/HEBP1/CAT/BLVRB/ ALAD/CA1/EPB41/SNCA/HMBS |

Figure S7. GSEA pathways for differentially expressed proteins at RA onset compared to 0-3 months pre-RA from the 7k panel


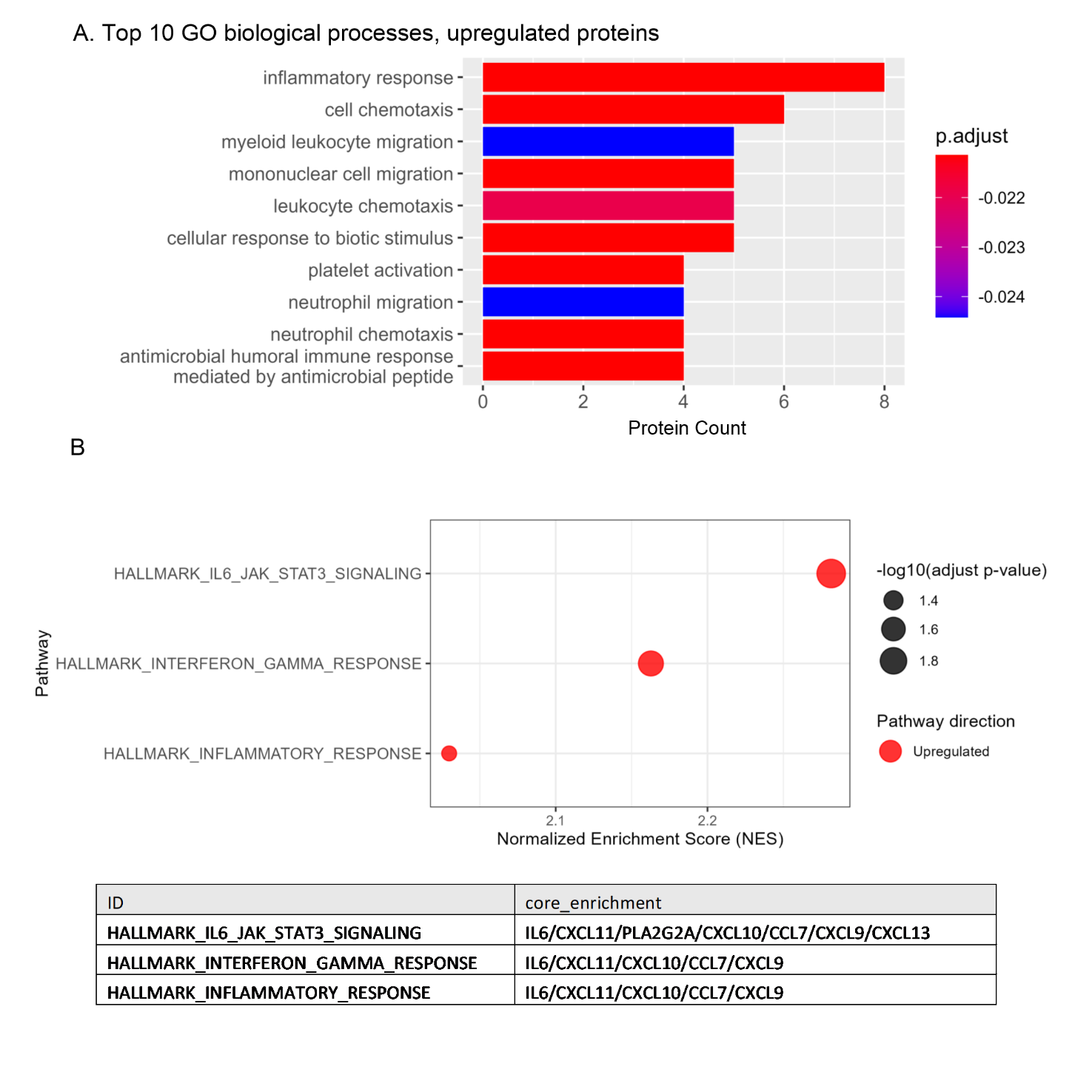
**Figure S8. Pathway analysis for differentially expressed proteins in serum from RA progressors compared to non-progressors regardless of treatment arm**

1. GO biological processes for upregulated proteins from the 7k panel
2. GSEA pathways for differentially expressed proteins from the 7k panel


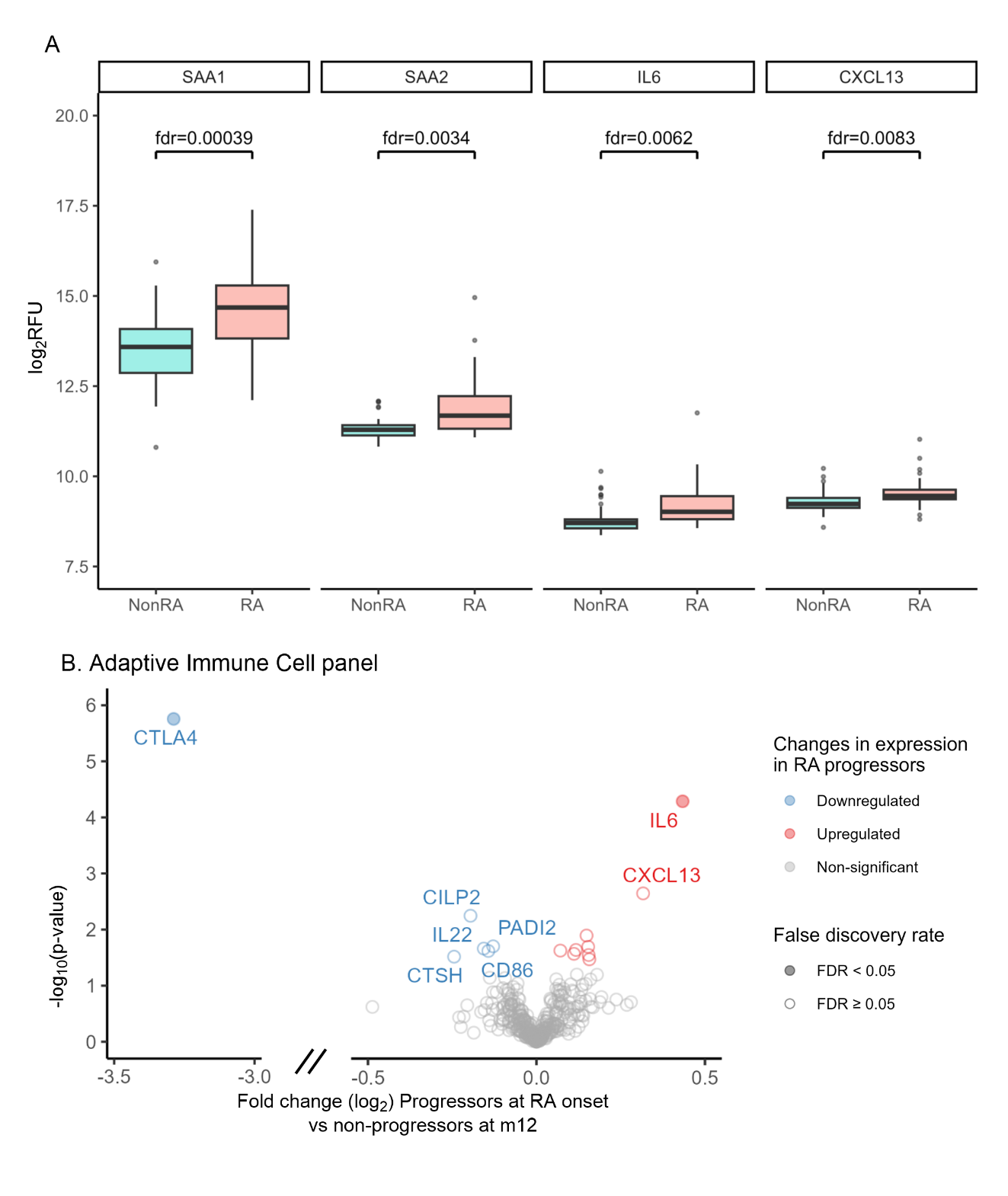


Figure S9. Differentially expressed proteins from the Inflammatory Mediators and Adaptive Immune Cell panel in RA progressors at RA onset compared to non-progressors at the end of treatment regardless of treatment arm

1. Boxplots for upregulated SAA1, SAA2, IL-6, CXCL13 from the Inflammatory Mediators panel
2. Volcano plot for Adaptive Immune Cell panel with differentially expressed downregulated CTLA4 and upregulated IL-6

Progressors at RA onset visit were compared to non-progressors at the end of treatment at month 12 regardless of treatment arm. Adjusted p-values/fdr in (A) were calculated from Welch’s t-test with unequal variances used at differential expression analysis in the Inflammatory Mediators panel. CTLA4=Cytotoxic T-lymphocyte protein 4; CXCL13=C-X-C motif chemokine 13; FDR=False Discovery Rate; IL-6=Interleukin-6; m12=Month 12; NonRA=Non-progressors at m12; RA=Progressors at RA onset; RFU=Relative Fluorescence Units; SAA1=Serum amyloid A-1 protein; SAA2=Serum amyloid A-2 protein.


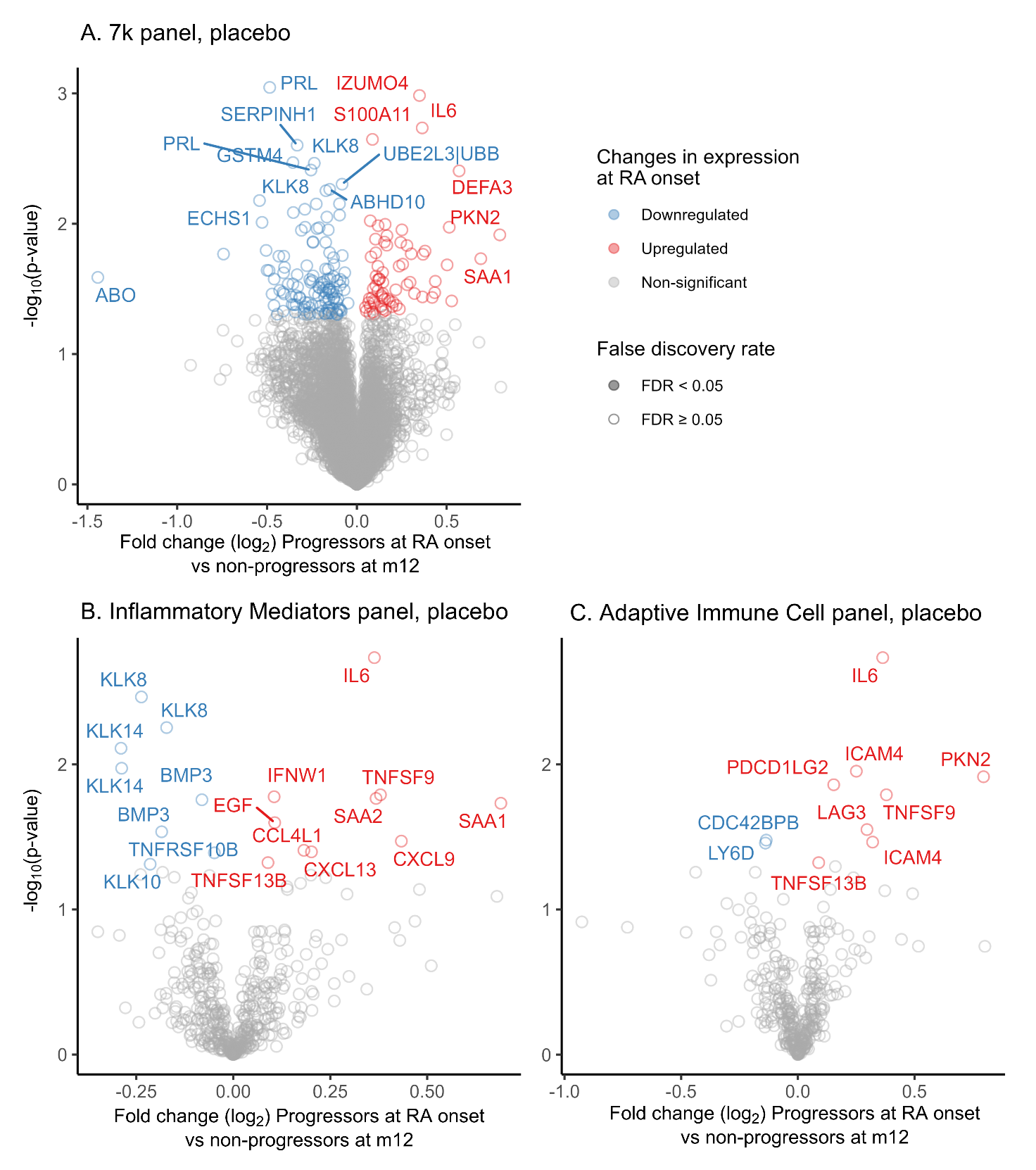


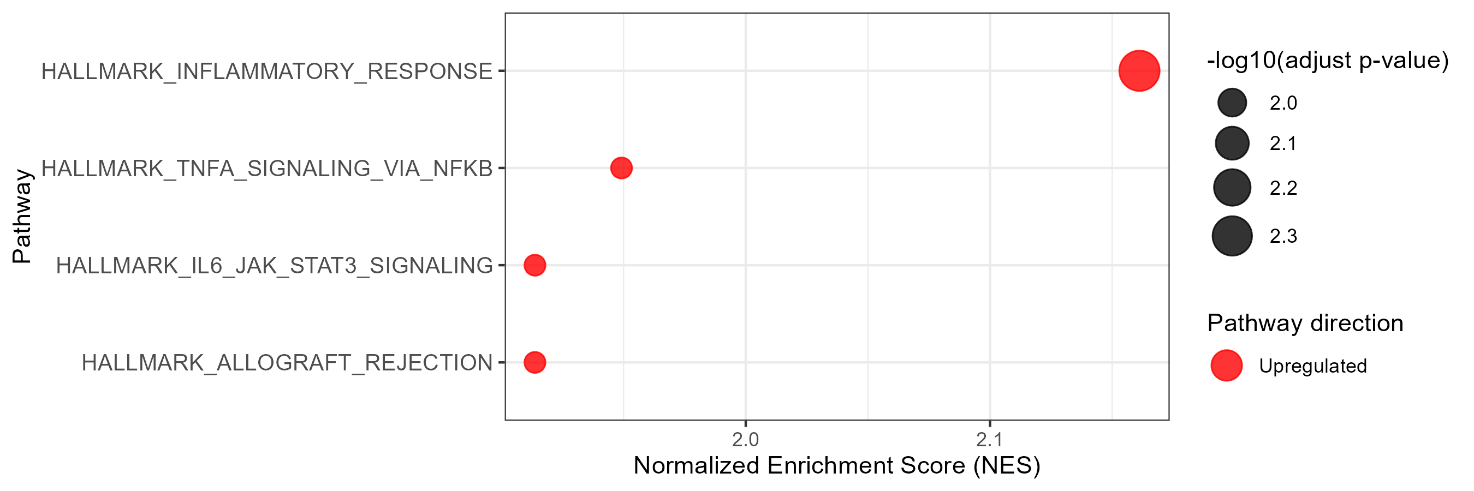


HALLMARK_INFLAMMATORY_RESPONSE (CXCL9/TNFSF9/IL6/ICAM4/TNFAIP6)

HALLMARK_TNFA_SIGNALING_VIA_NFKB (TNFSF9/CXCL3/IL6/TNFAIP6)

HALLMARK_ALLOGRAFT_REJECTION (CXCL9/IL6/SIT1/CXCL13)

HALLMARK_IL6_JAK_STAT3_SIGNALING (CXCL9/CXCL3/IL6/CXCL13)

D

**Figure S10. Pathway analysis for differentially expressed proteins in serum from RA progressors compared to non-progressors in the placebo arm**

1. Volcano plot for 7k panel with no differentially expressed proteins following Benjamini-Hochberg correction
2. Volcano plot for Inflammatory Mediators panel N=479 with no differentially expressed proteins following Benjamini-Hochberg correction
3. Volcano plot for Adaptive Immune Cell panel N=373 with no differentially expressed proteins following Benjamini-Hochberg correction
4. GSEA pathways for differentially expressed proteins from the 7k panel in placebo RA progressors at RA onset compared to placebo non-progressors at the end of treatment

FDR=False Discovery Rate; RA=Rheumatoid Arthritis.


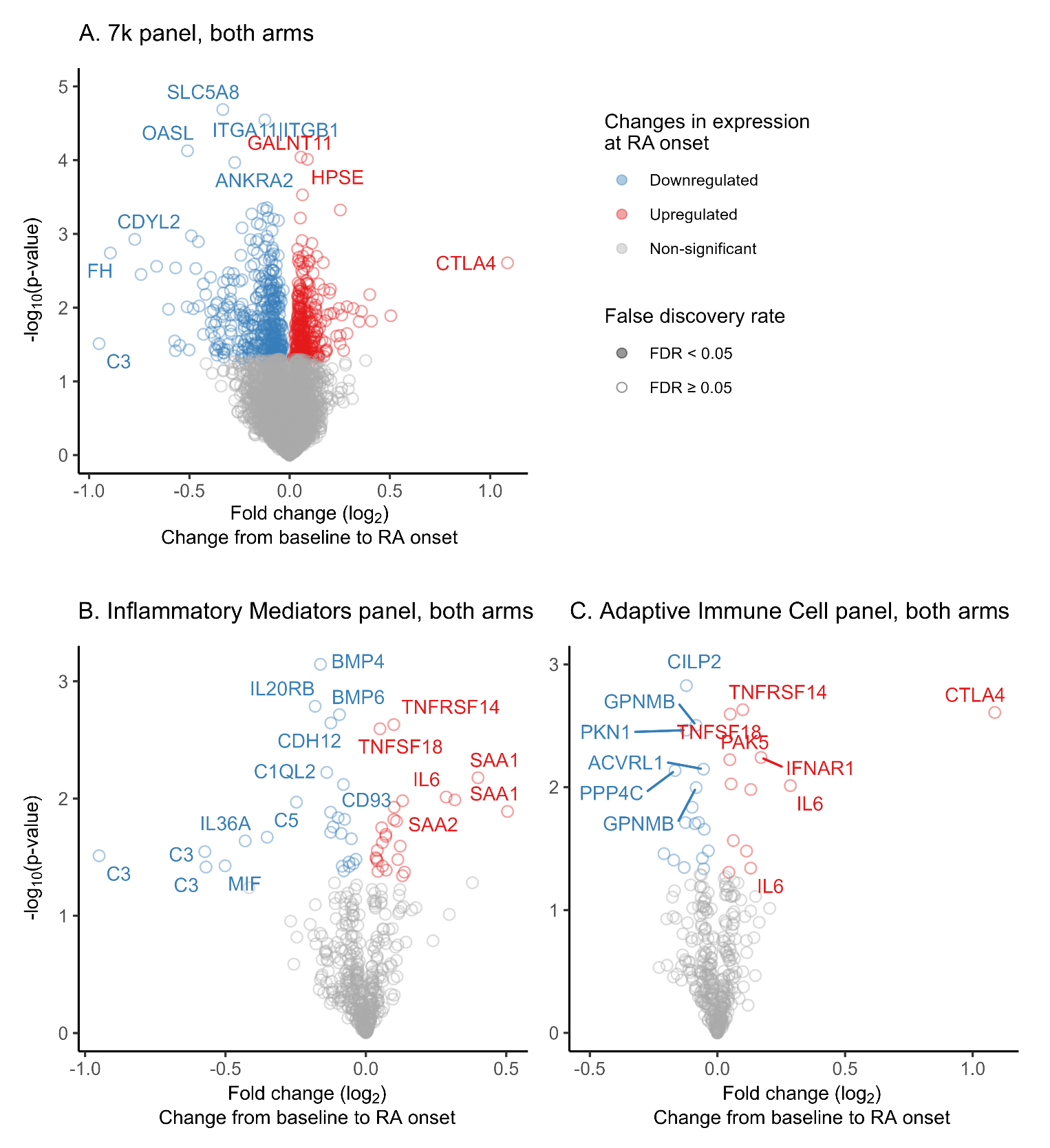


Figure S11. Changes from baseline to RA onset regardless of trial arm

1. Volcano plot for 7k panel with no differentially expressed proteins following Benjamini-Hochberg correction
2. Volcano plot for Inflammatory Mediators panel N=479 with no differentially expressed proteins following Benjamini-Hochberg correction
3. Volcano plot for Adaptive Immune Cell panel N=373 with no differentially expressed proteins following Benjamini-Hochberg correction

FDR=False Discovery Rate; RA=Rheumatoid Arthritis.


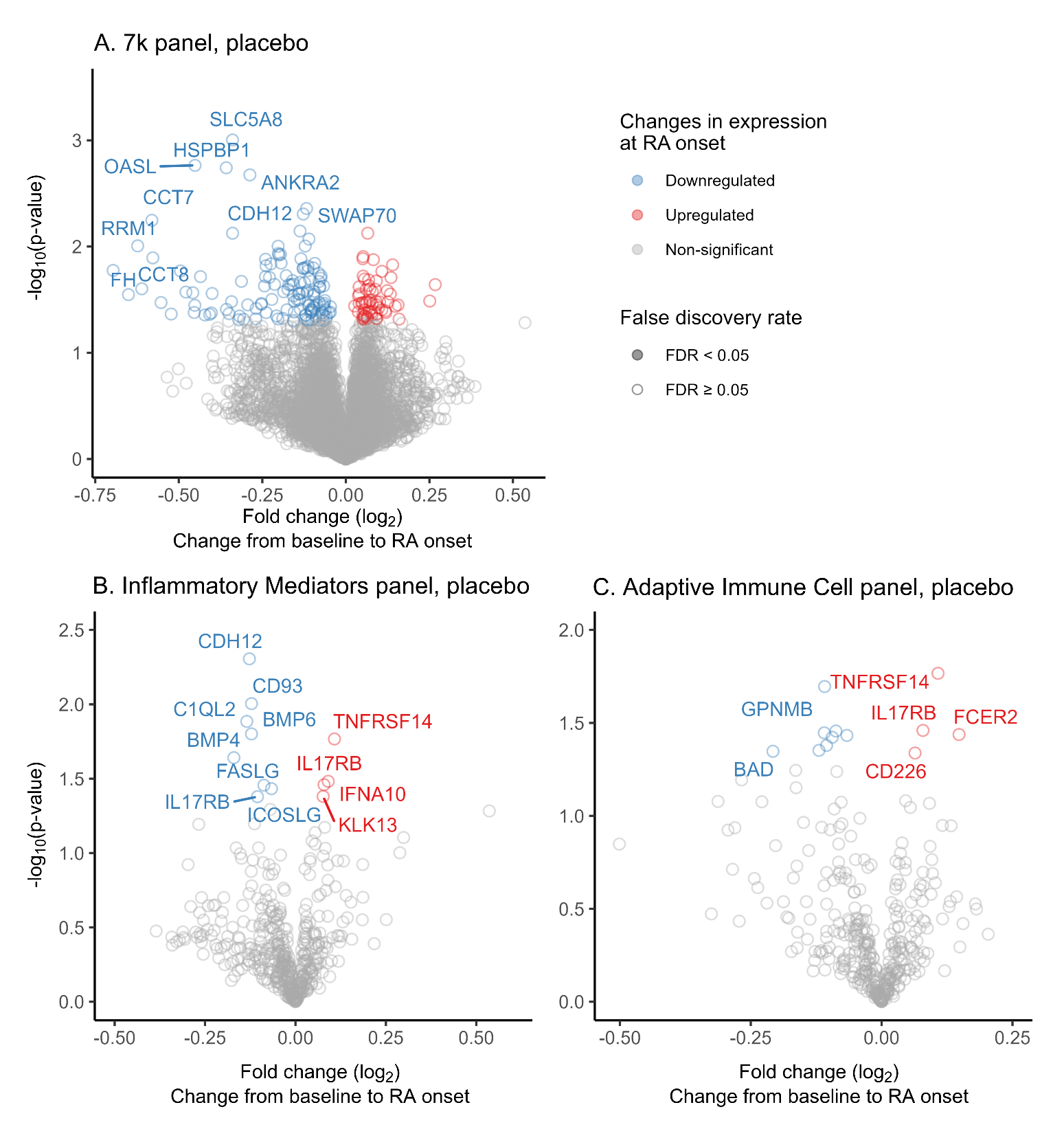


Figure S12. Changes from baseline to RA onset for placebo arm

1. Volcano plot for 7k panel with no differentially expressed proteins following Benjamini-Hochberg correction
2. Volcano plot for Inflammatory Mediators panel N=479 with no differentially expressed proteins following Benjamini-Hochberg correction
3. Volcano plot for Adaptive Immune Cell panel N=373 with no differentially expressed proteins following Benjamini-Hochberg correction

FDR=False Discovery Rate; RA=Rheumatoid Arthritis.


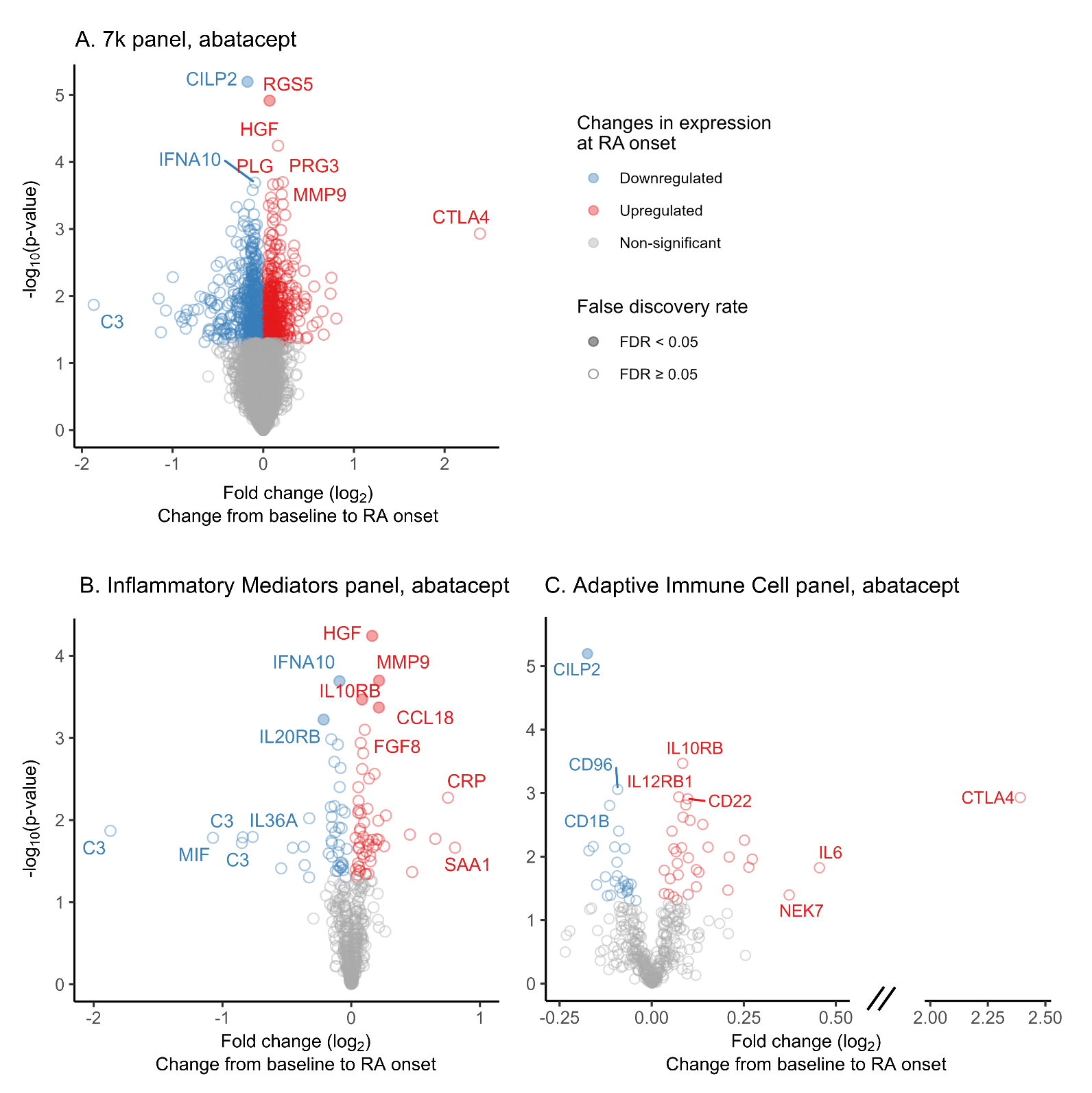


Figure S13. Changes from baseline to RA onset for abatacept arm

1. Volcano plot for 7k panel with downregulated CILP2 and upregulated RGS5 differentially expressed proteins following Benjamini-Hochberg correction
2. Volcano plot for Inflammatory Mediators panel N=479 with two downregulated IL20RB, IFNA10 and four upregulated differentially expressed proteins CCL18, HGF, MMP9, IL10RB following Benjamini-Hochberg correction
3. Volcano plot for Adaptive Immune Cell panel N=373 with downregulated differentially expressed CILP2 following Benjamini-Hochberg correction

CCL18=C-C motif chemokine 18; CILP2=Cartilage intermediate layer protein 2; FDR=False Discovery Rate; HGF= Hepatocyte growth factor; MMP9=Matrix metalloproteinase-9; IFNA10=Interferon alpha-10; IL10RB=Interleukin-10 receptor subunit beta; IL20RB=Interleukin-20 receptor subunit beta; RA=Rheumatoid Arthritis; RGS5=Regulator of G-protein signalling 5.


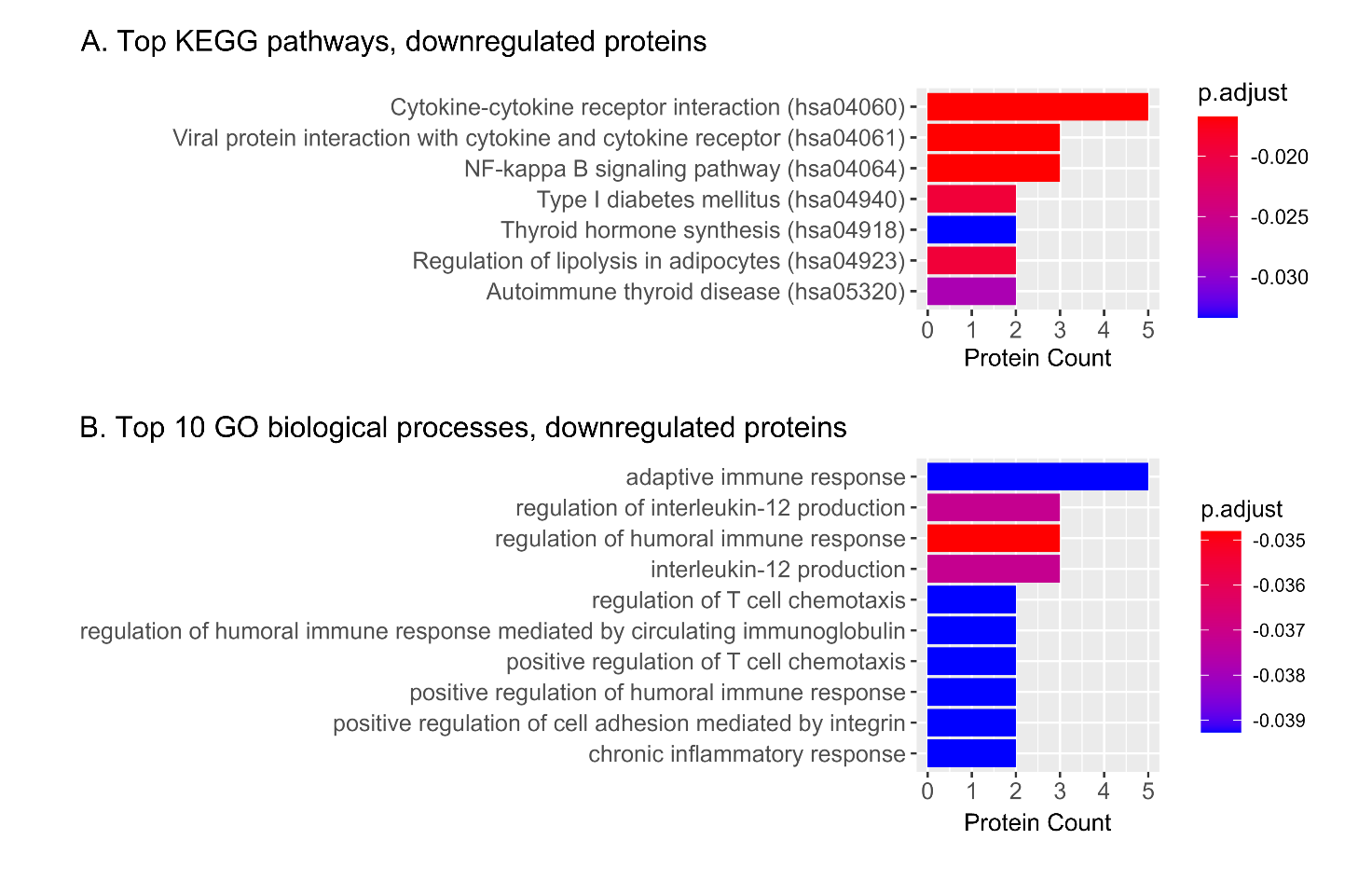

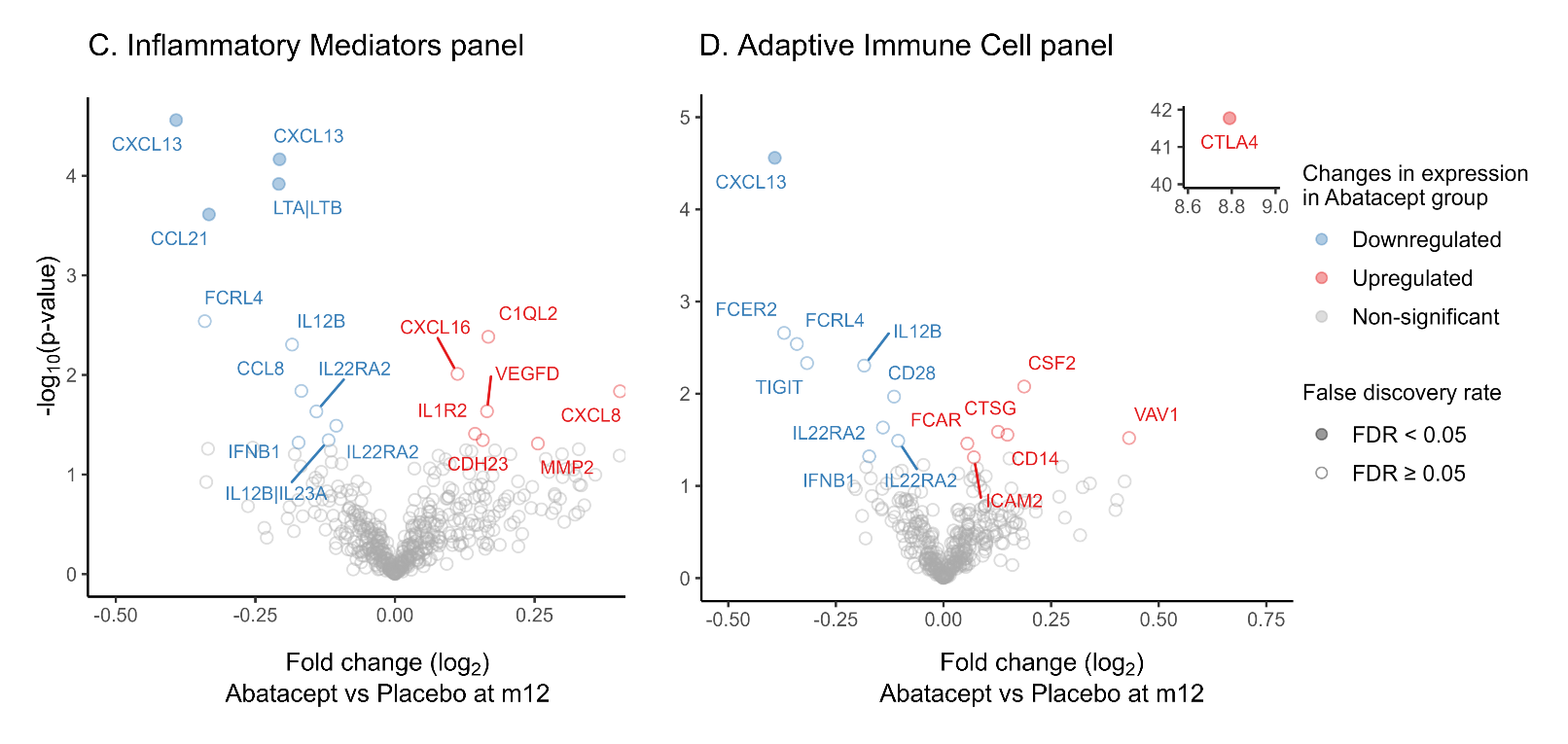
Figure S14. Pathway analysis for differentially expressed proteins in serum from participants randomised to abatacept compared to placebo at the end of treatment at month 12 regardless of outcome

1. KEGG pathways for downregulated proteins from the 7k panel.
2. GO biological processes for downregulated proteins from the 7k panel.
3. Volcano plot for Inflammatory Mediators panel N=479 with three downregulated differentially expressed CXCL13, CCL21 and LTA|LTB. Two correlated analytes target different epitope of CXCL13.
4. Volcano plot for Adaptive Immune Cell panel N=373 with two differentially expressed downregulated CXCL13 and upregulated CTLA4.

CTLA4=Cytotoxic T-lymphocyte protein 4; CCL21=C-C motif chemokine 21; CXCL13=C-X-C motif chemokine 13; FDR=False Discovery Rate; GO=Gene Ontology; KEGG=Kyoto Encyclopedia of Genes and Genomes; LTA|LTB=Lymphotoxin alpha1: beta2; m12=Month 12.

**Figure S15.** Pathway analysis for differentially expressed proteins in serum from **participants ‘on abatacept’ vs placebo**


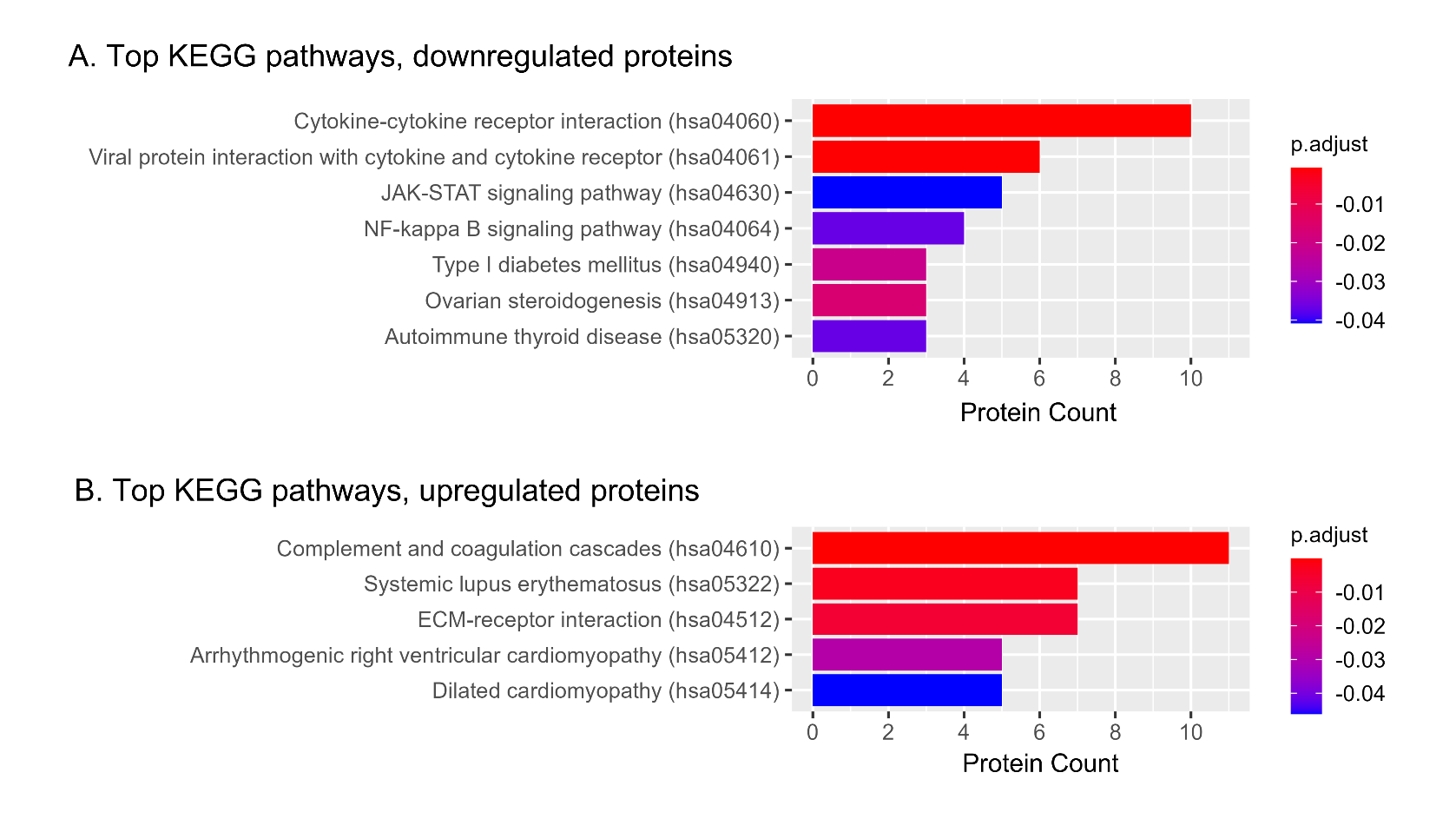

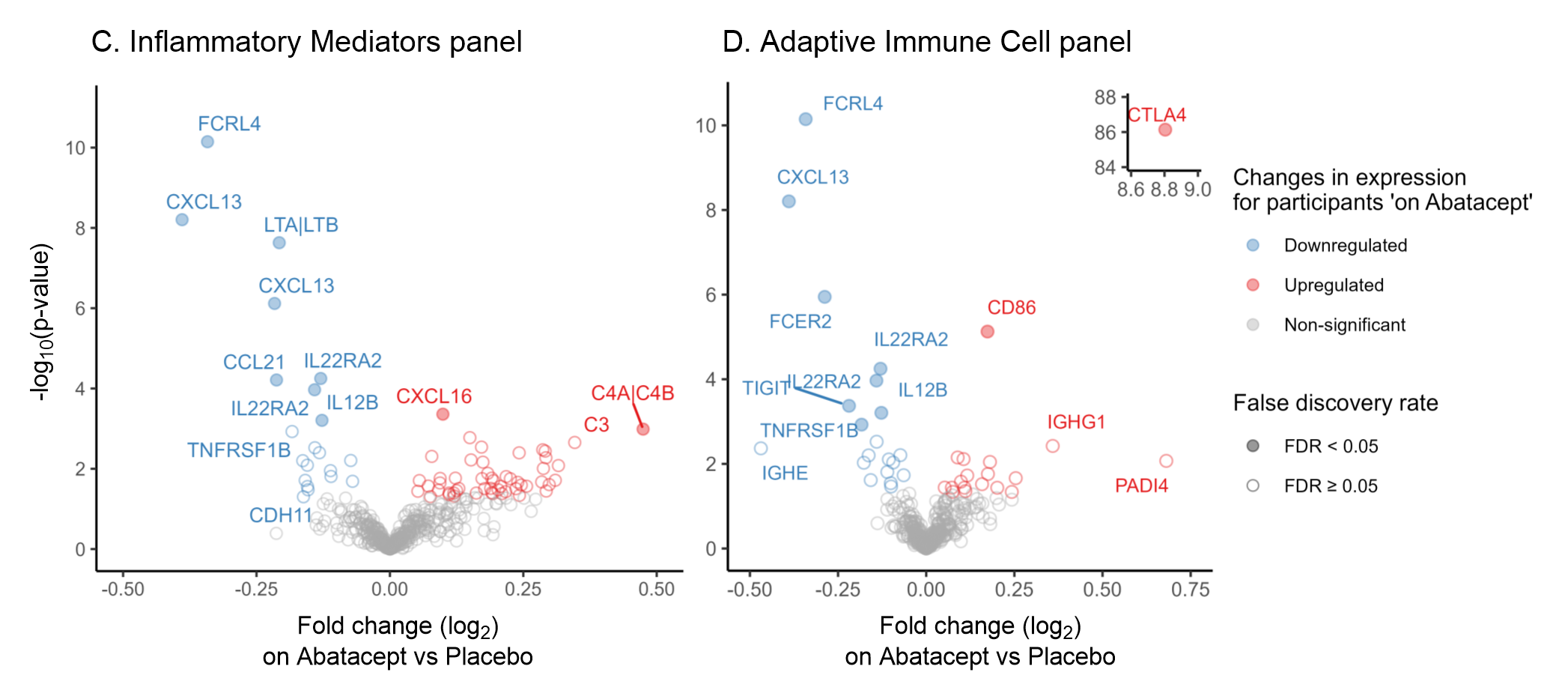


1. KEGG pathways for downregulated proteins from the 7k panel
2. KEGG pathways for upregulated proteins from the 7k panel
3. Volcano plot for Inflammatory Mediators panel N=479 with downregulated differentially expressed CXCL13, FCRL4, CCL21, IL22RA2, LTA|LTB, IL12B and two upregulated C4A|C4B and CXCL16.
4. Volcano plot for Adaptive Immune Cell panel N=373 with differentially expressed downregulated FCRL4, CXCL13, FCER2 (CD23), IL22RA2, TIGIT, IL12B, TNFRSF1B and upregulated CTLA4 and CD86.

‘On abatacept’ represents visits from baseline (excluding baseline) to the end of treatment at month 12. C4A|C4B= Complement C4; CTLA4=Cytotoxic T-lymphocyte protein 4; CCL21=C-C motif chemokine 21; CXCL13=C-X-C motif chemokine 13; CXCL16=C-X-C motif chemokine 16; FCER2 (CD23)=Low affinity immunoglobulin epsilon Fc receptor; FCRL4= Fc receptor-like protein 4; FDR=False Discovery Rate; GO=Gene Ontology; IL12B=Interleukin-12 subunit beta; IL22RA2=Interleukin-22 receptor subunit alpha-2; LTA|LTB=Lymphotoxin alpha1:beta2; TIGIT=T-cell immunoreceptor with Ig and ITIM domains; TNFRSF1B=Tumor necrosis factor receptor superfamily member 1B.


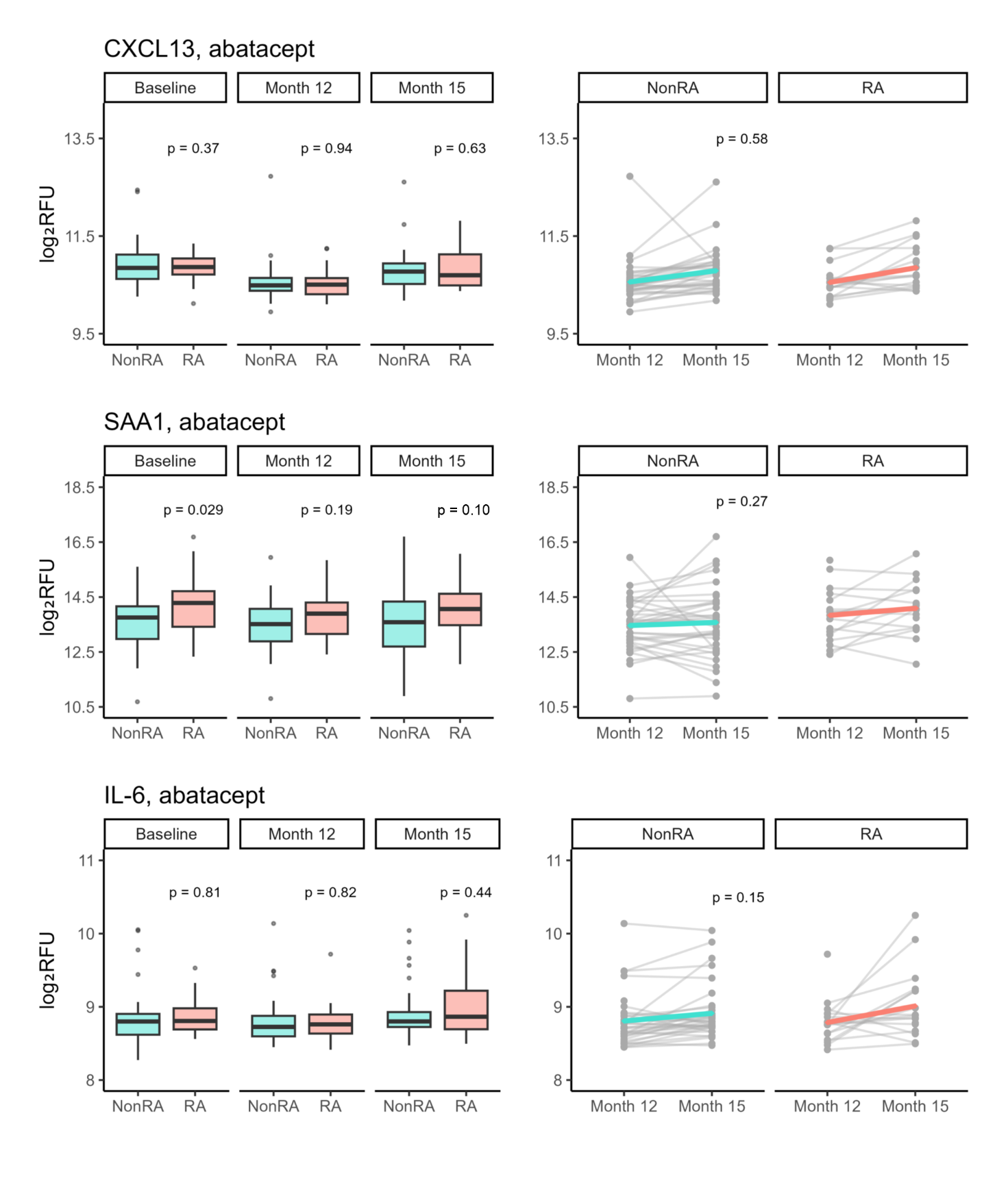


**Figure S16. Longitudinal analysis of expression of serum** **CXCL13, SAA1, and IL-6 in abatacept participants stratified by progression outcome**

Differences over time between abatacept RA progressors and abatacept non-progressors are shown in boxplots on the left. Differences in log2 fold change from month 12 (end of treatment) to month 15 between RA progressors and non-progressors in the abatacept group are shown in line plots on the right. Highlighted lines indicate group means and connected dots of individual observations. Statistical significance was determined using Welch’s t-test with unequal variances. CXCL13=C-X-C motif chemokine 13; IL-6=Interleukin-6; NonRA=Non-progressors; RA=Rheumatoid Arthritis progressors; RFU=Relative Fluorescence Units; SAA1=Serum amyloid A-1 protein


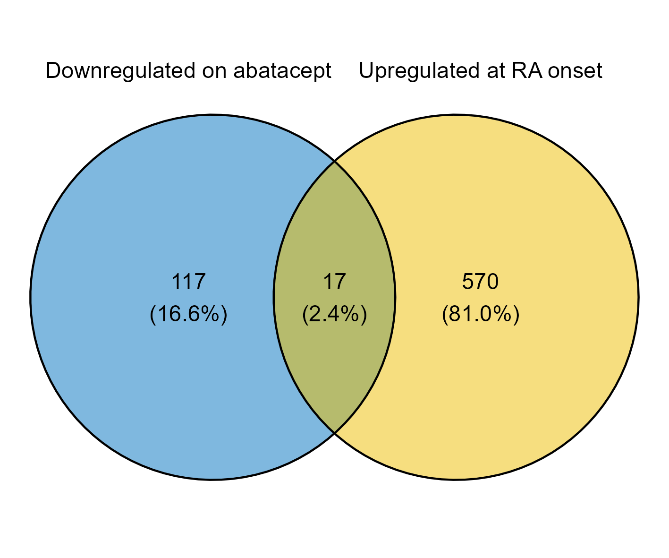
A

| **No** | **Symbol** | **Target Full Name** |
| --- | --- | --- |
| 1 | CLEC4G | C-type lectin domain family 4 member G |
| 2 | CXCL10 | C-X-C motif chemokine 10 |
| 3 | CXCL13 | C-X-C motif chemokine 13 |
| 4 | CXCL13 | C-X-C motif chemokine 13 |
| 5 | DLL1 | Delta-like protein 1 |
| 6 | FCER2 | Low affinity immunoglobulin epsilon Fc receptor |
| 7 | GLA | Alpha-galactosidase A |
| 8 | IL18BP | Interleukin-18-binding protein |
| 9 | KIR2DL5A | Killer cell immunoglobulin-like receptor 2DL5A |
| 10 | LGALS9 | Galectin-9 |
| 11 | NUDT9 | ADP-ribose pyrophosphatase, mitochondrial |
| 12 | SNPH | Syntaphilin |
| 13 | STATH | Statherin |
| 14 | TICAM2 | TIR domain-containing adapter molecule 2 |
| 15 | TIGIT | T-cell immunoreceptor with Ig and ITIM domains |
| 16 | TNFRSF1B | Tumor necrosis factor receptor superfamily member 1B |
| 17 | TXNDC5 | Thioredoxin domain-containing protein 5 |


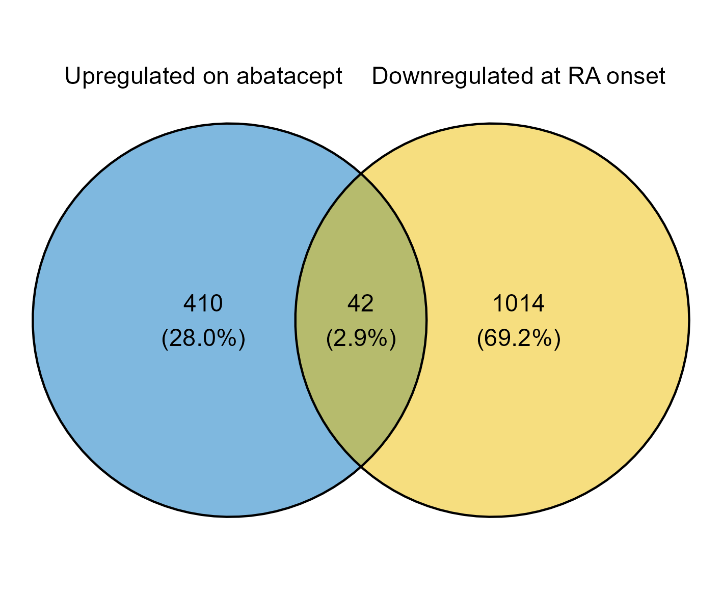
B

| **No** | **Symbol** | **Target Full Name** | **No** | **Symbol** | **Target Full Name** |
| --- | --- | --- | --- | --- | --- |
| 1 | ACHE | Acetylcholinesterase | 22 | H2AC11 | Histone H2A type 1 |
| 2 | ADAM23 | Disintegrin and metalloproteinase domain-containing protein 23 | 23 | H2BC21 | Histone H2B type 2-E |
| 3 | AFM | Afamin | 24 | HPGDS | Hematopoietic prostaglandin D synthase |
| 4 | AIF1 | Allograft inflammatory factor 1 | 25 | IHH | Indian hedgehog protein |
| 5 | ALB | Serum albumin | 26 | IRF2 | Interferon regulatory factor 2 |
| 6 | ANTXR2 | Anthrax toxin receptor 2 | 27 | ISLR2 | Immunoglobulin superfamily containing leucine-rich repeat protein 2 |
| 7 | ART3 | Ecto-ADP-ribosyltransferase 3 | 28 | ITGAV\|ITGB3 | Integrin alpha V beta 3 |
| 8 | BMP5 | Bone morphogenetic protein 5 | 29 | LY6G6C | Lymphocyte antigen 6 complex locus protein G6c |
| 9 | C1QL2 | Complement C1q-like protein 2 | 30 | MCAM | Melanoma-associated antigen MUC18 |
| 10 | C1QL3 | Complement C1q-like protein 3 | 31 | NCAM2 | Neural cell adhesion molecule 2 |
| 11 | CACNA2D3 | Voltage-dependent calcium channel subunit alpha-2/delta-3 | 32 | OMD | Osteomodulin |
| 12 | CHAD | Chondroadherin | 33 | PADI4 | Protein-arginine deiminase type-4 |
| 13 | CHL1 | Neural cell adhesion molecule L1-like protein | 34 | PCDHGA12 | Protocadherin gamma-A12 |
| 14 | CILP2 | Cartilage intermediate layer protein 2 | 35 | PDGFRL | Platelet-derived growth factor receptor-like protein |
| 15 | COL11A2 | Collagen alpha-2(XI) chain | 36 | POMGNT2 | Protein O-linked-mannose beta-1,4-N-acetylglucosaminyltransferase 2 |
| 16 | CTLA4 | Cytotoxic T-lymphocyte protein 4 | 37 | SMC3 | Structural maintenance of chromosomes protein 3 |
| 17 | CYBC1 | Uncharacterized protein C17orf62 | 38 | SULT6B1 | Sulfotransferase 6B1 |
| 18 | DNER | Delta and Notch-like epidermal growth factor-related receptor: Extracellular domain | 39 | TAGLN3 | Transgelin-3 |
| 19 | DPEP1 | Dipeptidase 1 | 40 | THSD1 | Thrombospondin type-1 domain-containing protein 1 |
| 20 | FAM110A | Protein FAM110A | 41 | TNXB | Tenascin-X |
| 21 | GXYLT1 | Glucoside xylosyltransferase 1 | 42 | WTAP | Pre-mRNA-splicing regulator WTAP |

**Figure S17. Venn diagram with intersection table for differentially expressed proteins associated with risk and response**

1. Downregulated proteins in participants on abatacept compared to upregulated proteins at RA onset
2. Upregulated proteins in participants on abatacept compared to downregulated proteins at RA onset

Differentially expressed proteins were considered from three panels of 7k, Inflammatory Mediators and Adaptive Immune Cell panels and defined by having unadjusted p-value less than 0.05. Upregulated and downregulated proteins at RA onset were identified with regards to three pre-RA time intervals defined in Figure 2A. There was no intersection for differentially expressed proteins with adjusted p-value less than 0.05.

**
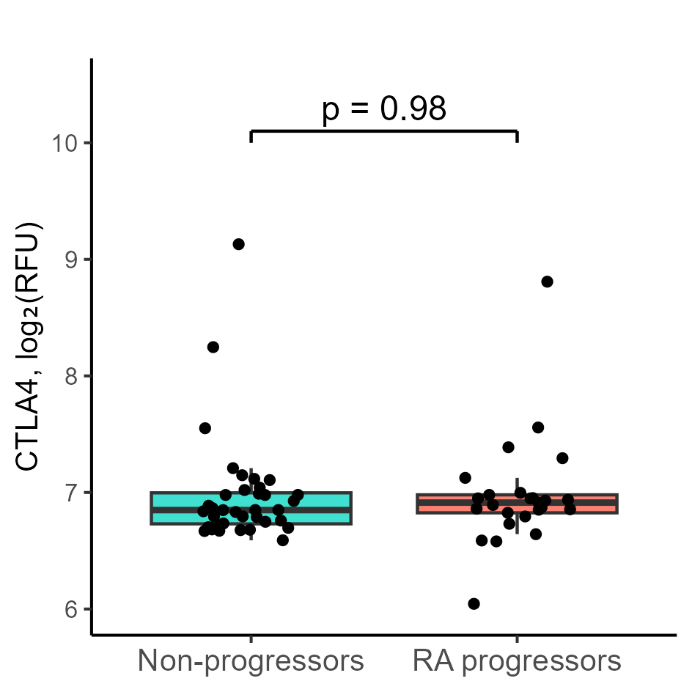
**

**Figure S18. Baseline CTLA4 (log2 scale) expression in placebo group stratified by progression outcome**

Statistical significance was determined using Welch’s 2 sample t-test with unequal variances. CTLA4=Cytotoxic T-lymphocyte protein 4; RA=Rheumatoid Arthritis; RFU=Relative Fluorescence Units.

REFERENCES

1. Candia J, Daya GN, Tanaka T, et al. Assessment of variability in the plasma 7k SomaScan proteomics assay. Sci Rep. 2022;12(1):17147.

2. SomaLogic. SomaScan Assay v4.1 Technical note SL00000572 Rev 4: 2023-03 2023 [Available from: <https://somalogic.com/>].

3. Candia J. SomaScan Bioinformatics: Normalization, Quality Control, and Assessment of Pre-Analytical Variation. bioRxiv. 2024:2024.02.09.579724.

Statistical Analysis Plan
